# Disabled cell density sensing leads to dysregulated cholesterol synthesis in glioblastoma

**DOI:** 10.18632/oncotarget.14740

**Published:** 2017-01-19

**Authors:** Diane M. Kambach, Alan S. Halim, A.Gesine Cauer, Qian Sun, Carlos A. Tristan, Orieta Celiku, Aparna H. Kesarwala, Uma Shankavaram, Eric Batchelor, Jayne M. Stommel

**Affiliations:** ^1^ Radiation Oncology Branch, National Cancer Institute, National Institutes of Health, Bethesda, MD 20892, USA; ^2^ Laboratory of Pathology, National Cancer Institute, National Institutes of Health, Bethesda, MD 20892, USA

**Keywords:** cholesterol metabolism, oxygen utilization, glioblastoma, pre-clinical cancer therapies, cell cycle

## Abstract

A hallmark of cellular transformation is the evasion of contact-dependent inhibition of growth. To find new therapeutic targets for glioblastoma, we looked for pathways that are inhibited by high cell density in astrocytes but not in glioma cells. Here we report that glioma cells have disabled the normal controls on cholesterol synthesis. At high cell density, astrocytes turn off cholesterol synthesis genes and have low cholesterol levels, but glioma cells keep this pathway on and maintain high cholesterol. Correspondingly, cholesterol pathway upregulation is associated with poor prognosis in glioblastoma patients. Densely-plated glioma cells increase oxygen consumption, aerobic glycolysis, and the pentose phosphate pathway to synthesize cholesterol, resulting in a decrease in reactive oxygen species, TCA cycle intermediates, and ATP. This constitutive cholesterol synthesis is controlled by the cell cycle, as it can be turned off by cyclin-dependent kinase inhibitors and it correlates with disabled cell cycle control though loss of p53 and RB. Finally, glioma cells, but not astrocytes, are sensitive to cholesterol synthesis inhibition downstream of the mevalonate pathway, suggesting that specifically targeting cholesterol synthesis might be an effective treatment for glioblastoma.

## INTRODUCTION

Malignant gliomas are highly lethal adult brain tumors with glial features, likely derived from neural stem cells, glial progenitors, or astrocytes [1–3]. The most common and lethal malignant glioma is WHO grade IV glioblastoma, or GBM, which has a median survival of 15 months with current standard-of-care therapy consisting of surgery, radiation, and temozolomide [4]. While the bulk of these tumors can be removed surgically, total resection is impossible due to the highly invasive and infiltrative nature of the tumor cells. These tumors are also highly resistant to cytotoxic therapies: adjuvant treatment with temozolomide only improves survival 2.5 months beyond radiation and surgery alone [4]. Extensive genomic characterization by The Cancer Genome Atlas (TCGA) has revealed that the most frequently altered pathways in GBM are the RTK/PI3K/MAPK (90% of tumors), p53 (86%), and Rb pathways (79%) [5]. Of these, the RTK/PI3K/MAPK axis is the most targetable with state-of-the-art therapeutics, yet these drugs have not significantly improved patient survival beyond that of the current standard-of-care [4, 6, 7]. For example, despite the high prevalence of EGFR alterations in GBM (57%) [5], EGFR targeted therapies have thus far uniformly failed in the clinic [7, 8]. Therapies targeting other signaling molecules in this pathway have fared no better: multiple clinical trials have failed to demonstrate significantly extended patient survival with inhibitors of PDGFR, mTOR, VEGF, or VEGFR [6]. There are multiple possible explanations for the failure of these drugs, including signaling pathway redundancy [9], genomic heterogeneity among tumor cells [10, 11], and blood-brain barrier protection of infiltrative cells. Recent advances in glioma cell culture and genomics have accelerated the discovery of new pathways critical for the growth and survival of these cells. Specifically, patient-derived tumor stem-like cells grown in neural stem cell culture conditions retain genomic characteristics of primary patient tumors and form glioblastomas in orthotopic mouse models, thus providing a superior model system for studying GBM biology [12, 13]. Moreover, the wealth of copy number, mutation, and gene expression information in the more than 500 patient tumors profiled by the TCGA has stimulated the study of clinically-relevant tumor biology in the laboratory [5]. Thus, there is reason to be optimistic that new drug targets will be discovered.

Cholesterol is critical for cell viability and function. It is an important component of the plasma membrane and lipid rafts and is a precursor for steroid hormones, bile acids, and Vitamin D. Cholesterol can also act as a signaling molecule in its own right, for example, through binding and activating estrogen-related receptor alpha (ESRRA) [14]. Furthermore, oxygenated products of cholesterol, oxysterols, activate liver X receptor alpha (LXRA) [15] and inhibit estrogen receptor [16]. Cholesterol metabolism in the brain is unique compared to other organs. Because cholesterol cannot cross the blood-brain barrier, the brain must synthesize it *de novo* through the mevalonate and Bloch and Kandutsch-Russell pathways [17–19]. This is in contrast with other organs that can obtain dietary cholesterol from the bloodstream via delivery by the low density lipoprotein receptor (LDLR). Despite the requirement for the brain to synthesize cholesterol *de novo*, this organ contains 23% of all the cholesterol in the body [20]. Much of this cholesterol is in myelin sheaths, which are formed by oligodendrocytes to insulate axons. It is also a major component of synaptic vesicles and is critical for their formation and function [21]. Defects in cholesterol metabolism are central to some neurological disorders, such as Niemann-Pick type C disease, and are implicated in Alzheimer's disease and age-related cognitive decline [22]. High serum cholesterol levels are also associated with increased risk for many cancers [23], but thus far, clinical trials with HMG-CoA Reductase inhibitors (statins) have had mixed results [24].

Seminal studies in cancer cell biology in the 1960s showed that a fundamental property of tumorigenic cells is the evasion of contact-dependent inhibition of growth. Normal mouse fibroblasts can be coaxed into bypassing contact inhibition through repeated plating at high cell density [25]. These transformed cells can attain higher densities than normal fibroblasts, and can form subcutaneous tumors in mice [25, 26]. This suggests that normal cells have mechanisms for detecting when they have reached the appropriate density and for mounting the correct cellular response. Later, it was shown that high cell density confers resistance to cytotoxic cancer therapeutics such as anthracycline antibiotics, vinca alkaloids, taxanes, nitrosureas, and bleomycin [27–29].

Because of the important roles cell density plays in cancer cell growth and therapeutic response, we compared the metabolism of high and low density normal astrocytes to high and low density GBM patient-derived tumor stem-like cells. We found that at high cell density, normal astrocytes turn off cholesterol synthesis pathways and decrease cholesterol while glioblastoma cells ignore density cues and maintain cholesterol synthesis. Increased expression of genes in the cholesterol biosynthetic pathway correlates with poor prognosis in GBM patients irrespective of molecular sub-class, age, or *MGMT* status. High density glioblastoma cells increase oxygen consumption, aerobic glycolysis, and the pentose phosphate pathway to provide substrates for cholesterol synthesis, while simultaneously decreasing mitochondrial respiration. The appropriate regulation of cholesterol synthesis requires intact cell cycle control, as immortalized astrocytes lacking p53 and Rb no longer inhibit cholesterol synthesis at high density, and glioma cells arrested with CDK inhibitors have lower cholesterol. Finally, we found that glioma cells, but not normal astrocytes, are sensitive to shutting down cholesterol synthesis through pharmacological inhibition of lanosterol synthase or CYP51A1 in a density-dependent manner. These data suggest that cholesterol synthesis inhibition could be an important therapy for glioblastoma patients.

## RESULTS

### Normal astrocytes turn off cholesterol synthesis pathways at high cell density but glioma cells keep them active

**Figure 1:**
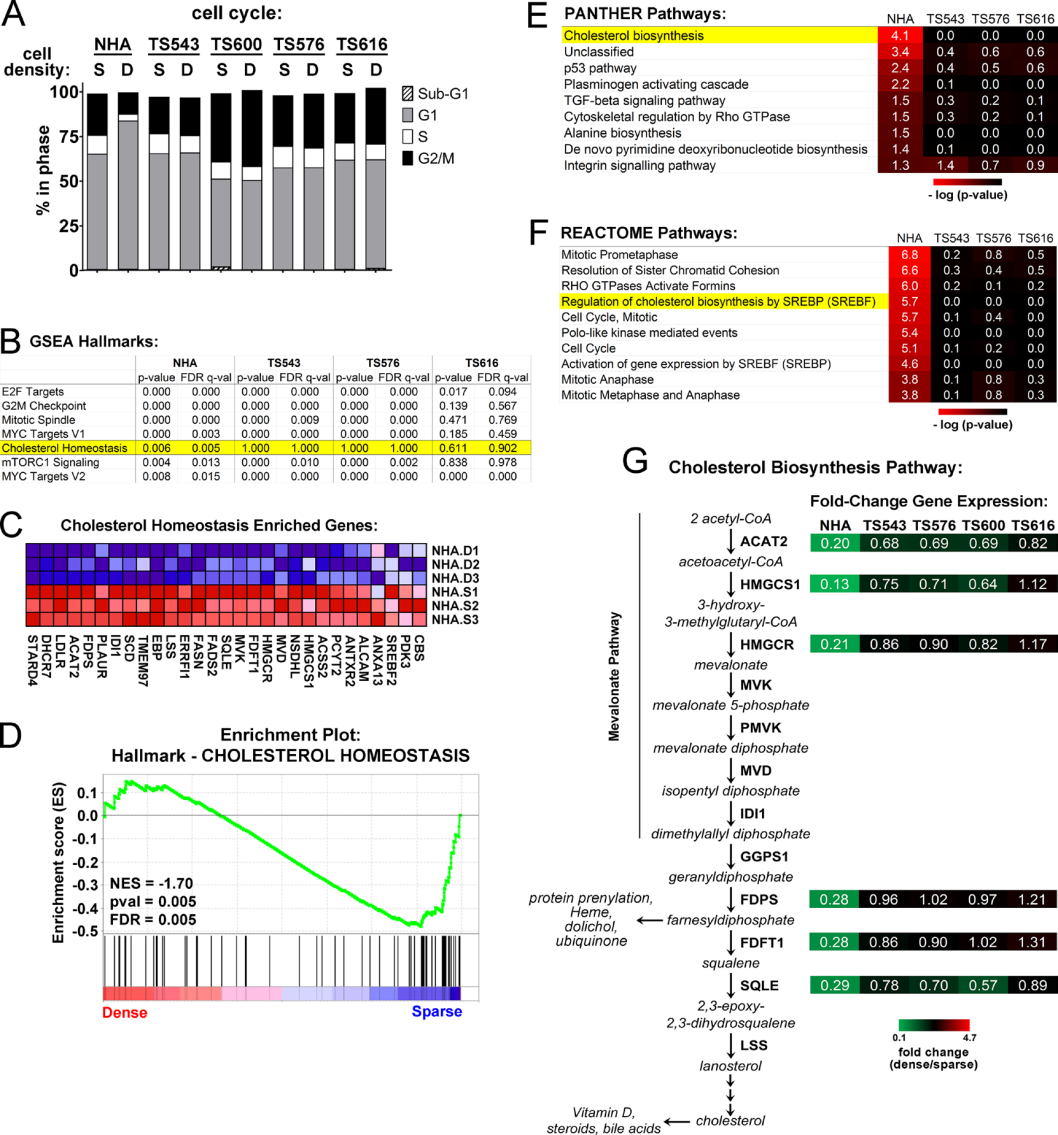
Cholesterol biosynthesis pathways are dysregulated in glioma cells plated at high density (**A**) Cell cycle analysis of sparse (S = 15,625 cells/cm^2^) and dense (D = 93,750 cells/cm^2^) cells. Shown is the average of 3 biological replicates. (**B**) Gene Set Enrichment Analysis (GSEA) for sparse and dense NHA and TS glioma cells. The top scoring Hallmarks for genes downregulated in dense NHAs and corresponding scores for the TS cells are shown. (**C**) Enriched genes for the GSEA Hallmark, “Cholesterol Homeostasis.” Three biological replicates for NHA dense (D) and sparse (S) are shown. (**D**) NHA cells GSEA Enrichment Plot for the Hallmark, “Cholesterol Homeostasis.” NES = Normalized Enrichment Score. (**E**) PANTHER Pathway classification for density-dependent genes in NHA and glioma TS cells. Significant pathways for genes downregulated in dense NHAs and corresponding *p*-values for the TS cells are shown. (**F**) REACTOME Pathway classification for density-dependent genes in NHA and glioma TS cells. The top ten significant pathways for genes downregulated in NHAs and corresponding *p*-values for the TS cells are shown. (**G**) Relative gene expression for select enzymes in the mevalonate and cholesterol biosynthesis pathways. Shown are gene expression values derived from quantitative real time PCR normalized to GAPDH and expressed as fold change of dense over sparse. Values are the mean of at least 3 biological replicates (see Supplementary Figure 1E for details).

Early fundamental studies in cancer cell biology showed that high cell density leads to cell transformation and drug resistance. We examined whether tumor stem-like cells derived from GBM patient tumors and maintained in neural stem cell medium (hereafter referred to as glioma tumor sphere (TS) lines [10, 30]) exhibit these hallmarks of transformation by continuing to proliferate at high cell densities. We found that while normal human astrocytes (NHA) arrested in G1 at high density, four different glioma TS lines, TS543, TS600, TS576, and TS616 all continued cycling (Figure 1A). To find pathways that may have been altered in the loss of contact inhibition, we compared gene expression in sparse and dense glioma TS cells and normal astrocytes. Overall, cells did not cluster by cell density but instead into two subgroups of normal and cancer (Supplementary Figure 1A). Nonetheless, when we compared gene sets specifically enriched in either sparse or dense cells using Gene Set Enrichment Analysis (GSEA), we observed that “Cholesterol Homeostasis” was significantly regulated by cell density in normal astrocytes but not in any of the glioma TS cells (Figure 1B–1D). In addition, “Cholesterol biosynthesis” was significantly downregulated only in dense NHAs but not dense glioma TS cells using PANTHER gene list analysis [31] (*p* = 7.40E-05, Figure 1E) and “Regulation of cholesterol biosynthesis by SREBP” was significantly downregulated in dense NHAs but not dense glioma TS cells in the REACTOME pathway database [32] (*p* = 1.90E-06, FDR *q* = 3.73E-04, Figure 1F). The NHAs grow as an adherent monolayer and in different culture medium than the glioma TS lines, which can grow either as suspended spheroids or as an adherent monolayer on laminin [13]. To validate that the differential regulation of the cholesterol biosynthetic pathway was not a result of different growth modes and culture media for the NHAs and cancer cells, we performed quantitative real time PCR on cDNAs derived from NHAs and 4 different glioma TS lines all grown in TS cell medium and adherent on laminin. Genes in the mevalonate pathway (*ACAT2, HMGCS1*, and *HMGCR*) and downstream in the cholesterol biosynthetic pathway (*FDPS, FDFT1*, and *SQLE*), were all downregulated in dense NHAs but not dense glioma TS lines (Figure 1G and Supplementary Figure 1E). Genes involved in the regulation of the mevalonate pathway (*INSIG1* and *SREBF2* but not *SREBF1*) were also downregulated in dense NHAs but not glioma cells (Supplementary Figure 1F). In contrast, while the lipoprotein transporter *APOE* was variably regulated by density across cell lines, the cholesterol efflux pump *ABCA1* was significantly upregulated in both the normal and tumor lines at high densities (Supplementary Figure 1F). Interestingly, neither of two colon cancer cell lines (HT29, HCT116) and only 1 of 2 lung cancer cell lines (NCI-H522, NCI-H3255) had constitutively activated mevalonate and cholesterol synthesis gene expression, suggesting that this might be a specific adaptation glioma cells acquire to keep cholesterol levels high when the blood-brain barrier blocks the uptake of dietary cholesterol from circulation (Supplementary Figure 1G).

### Upregulation of the mevalonate and cholesterol synthesis pathways is associated with poor patient survival in GBM

**Figure 2:**
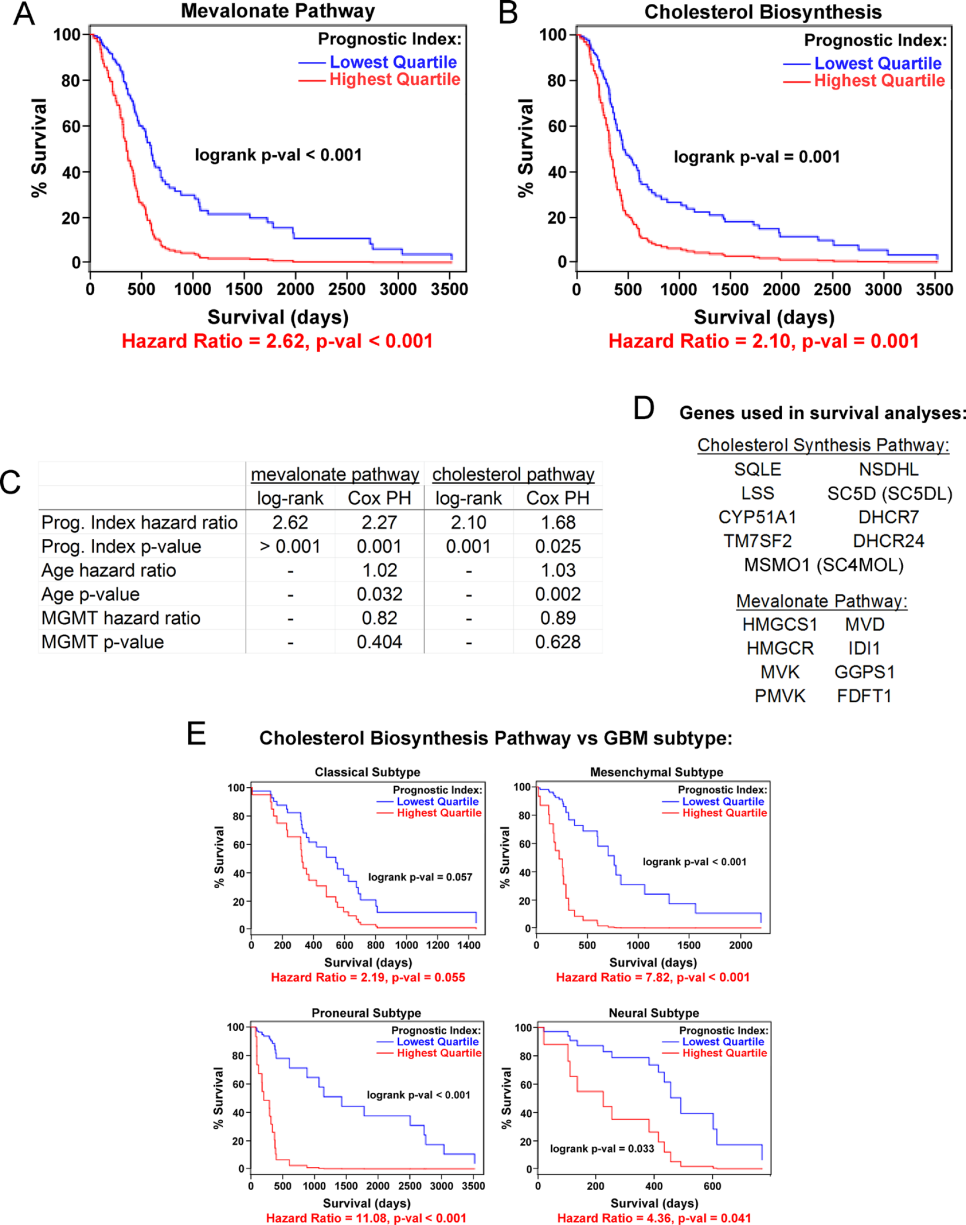
Cholesterol pathway upregulation is associated with poor prognosis for GBM patients Kaplan-Meier curves for expression of mevalonate (**A**) and cholesterol (**B**) biosynthesis pathway genes as a combined group, stratified by lowest vs. highest quartile gene expression. Data are from the TCGA. (**C**) Comparison of log-rank univariate vs. Cox proportional hazards (PH) multivariate modeling of prognostic indices for the mevalonate and cholesterol synthesis pathways. Cox PH modeling was adjusted for age and *MGMT* methylation status in addition to gene expression covariates. (**D**) Gene lists for survival curve data. (**E**) Kaplan-Meier curves for each of four GBM molecular subtypes [36] comparing lowest and highest quartile of cholesterol biosynthesis pathway gene expression.

We next asked whether the expression of cholesterol synthesis genes is associated with glioblastoma patient survival. Using the Glioblastoma Bio Discovery Portal [33], we examined clinical outcomes of 173 patients in the TCGA dataset with simultaneous overexpression of multiple genes in the mevalonate and cholesterol biosynthesis pathways (see Figure 1G and Supplementary Figure 2 for pathway diagrams). Patients in the highest gene expression quartile for both pathways have significantly shorter survival times than those in the lowest expression quartile (Hazard Ratio 2.62, *p-value* < 0.001 for mevalonate; Hazard Ratio 2.10, *p-value* = 0.001 for cholesterol, Figure 2A–2D). The hazard ratios for mevalonate and cholesterol pathway are significant in a multivariate Cos PH analysis adjusting for the known GBM prognostic indicators age and *MGMT* status [34, 35], suggesting that the correlation of these pathways with patient survival is independent of these factors (Figure 2C). Interestingly, upregulation of cholesterol synthesis genes was associated with decreased survival across all four glioblastoma molecular subtypes [36], though this signature predicted the worst prognosis in the mesenchymal and proneural subtypes (Figure 2E). In contrast, overall survival is not associated with the overexpression of any individual gene in either of these pathways (Supplementary Figure 3A, 3B), and on average, none of the genes individually is overexpressed relative to normal brain (Supplementary Figure 3C, 3D), as might be expected for an enzymatic pathway in which many of the steps might be rate-limiting. Together, these data suggest that therapies disrupting the cholesterol synthesis pathway at any of multiple points might be beneficial across a wide range of GBM patients.

### Glioma cells increase oxygen consumption to make cholesterol at high cell densities

**Figure 3:**
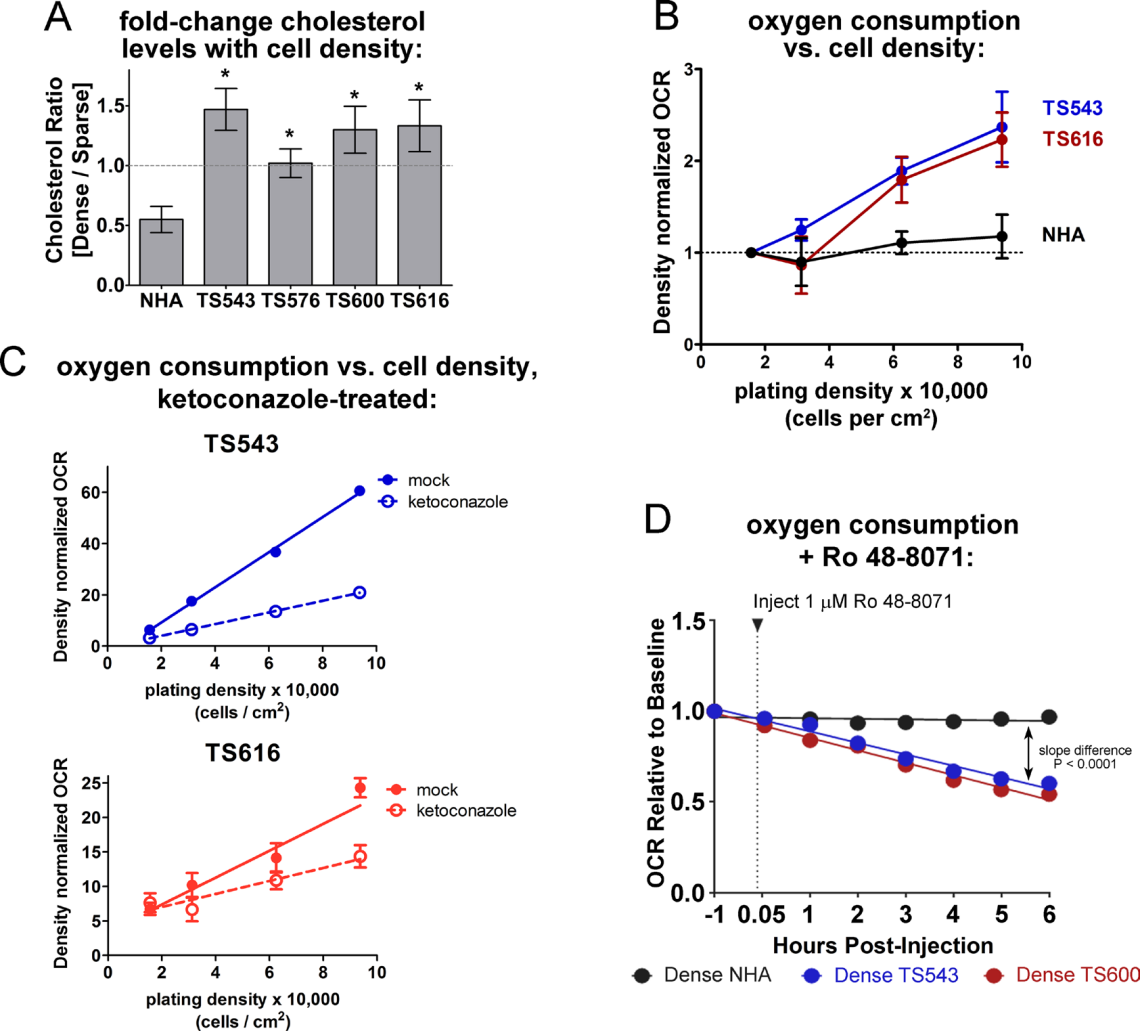
Dense glioma cells use oxygen to maintain cholesterol synthesis (**A**) Cholesterol quantitation in NHA and glioma TS cells, expressed as the ratio of dense (93,750 cells/cm^2^) over sparse (15,625 cells/cm^2^). Cholesterol was measured with the Amplex Red Cholesterol Assay Kit. *p < 0.05, two-tailed *t-test* vs. NHA. Bars = SEM for at least 3 biological replicates. (**B**) OCR (oxygen consumption rate) in NHA and glioma TS cells plated at the indicated densities. OCR was normalized to DNA content to control for cell number and to sparse OCR to show fold change with increasing density. Bars = SEM for at least 3 biological replicates. (**C**) OCR in glioma TS543 and TS616 cells plated at the indicated densities. Cells were treated with 5 μM ketoconazole for 6 hours prior to OCR measurements. OCR is normalized to DNA content to control for cell number. Bars = SD. Data shown are representative of 2 biological replicates. (**D**) OCR in mock- and Ro 48-8071-treated dense TS543 and TS600 glioma TS cells and NHAs. OCR is normalized to DNA content to control for cell number.

Because the glioma TS cells fail to switch off the expression of cholesterol synthesis genes at high density, we asked whether they also maintain cholesterol levels. Consistent with the cholesterol pathway gene expression shown in Figure 1, we observed that NHAs had a significant decrease in cholesterol at high density while the glioma TS cells maintained or increased it (Figure 3A and Supplementary Figure 4A). Cholesterol synthesis is an oxygen-intensive metabolic process: eleven molecules of O_2_ are required to make one molecule of cholesterol [37] (Supplementary Figure 2), and under conditions in which oxidative phosphorylation is inhibited, about 25% of oxygen utilization in *S. cerevisiae* goes toward sterol biosynthesis [38]. Dense glioma cells also increased their oxygen consumption rates (OCR) relative to sparse while the OCR of the NHAs remained the same in both low and high density cells (Figure 3B and Supplementary Figure 4B). If the increased oxygen consumption in dense glioma TS cells is used for cholesterol synthesis, inhibiting this pathway should lower oxygen consumption in these cells. To test this hypothesis, we treated glioma TS cells with two different pharmacological inhibitors of the cholesterol synthesis pathway: ketoconazole, an FDA-approved antifungal that blocks the demethylation of lanosterol by CYP51A1 [39], and Ro 48-8071, which inhibits the formation of lanosterol by the oxidosqualene cyclase LSS [40] (Supplementary Figure 2). A six-hour treatment with either drug was sufficient to reduce cholesterol levels in glioma TS cells (Supplementary Figure 4C). Ketoconazole treatment reduced oxygen consumption in glioma TS cells in both a time- and density-dependent manner, with the greatest effect in dense cells (Figure 3C and Supplementary Figure 4D). Like ketoconazole, Ro 48-8071 decreased oxygen consumption in dense TS543 or TS600 cells but much less so in the normal astrocytes (Figure 3D). These data suggest that an important use of oxygen in dense glioma cells, but not normal astrocytes, is cholesterol synthesis.

### Glioma cells downregulate the TCA cycle to make cholesterol at high cell densities

**Figure 4:**
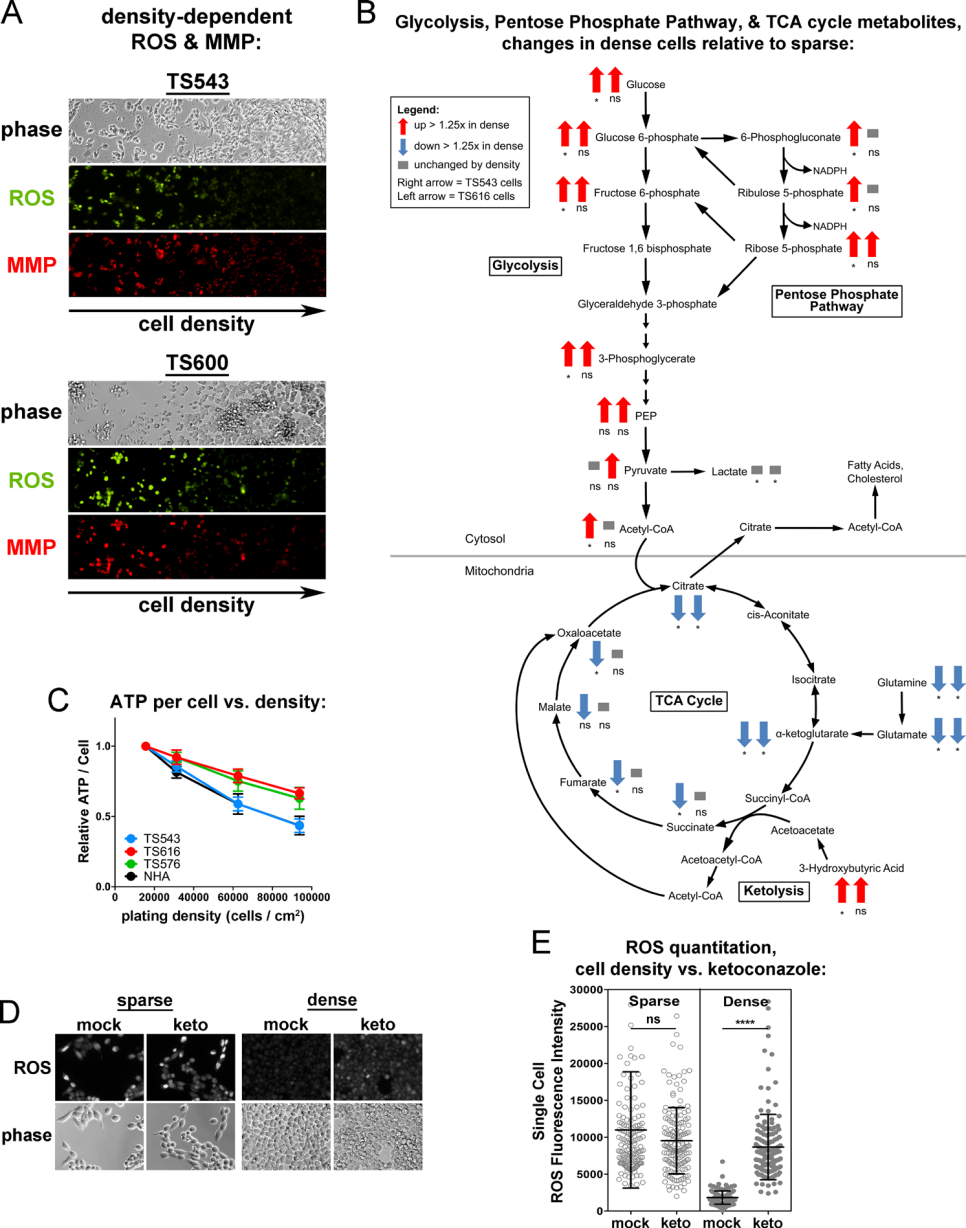
Dense cells have decreased mitochondrial respiratory activity and TCA cycle compared to sparse (**A**) ROS and mitochondrial membrane potential (MMP) in TS543 and TS600 cells cultured on a gradient of increasing cell density. (**B**) Density-dependent changes in glycolysis, pentose phosphate pathway, and TCA cycle metabolites in TS543 and TS616 cells. Arrows indicate > 1.25-fold increase (up red arrow) or > 1.25-fold decrease (down blue arrow) in dense cells. Left arrow = TS543 cells, right arrow = TS616. *p ≤ 0.1, **p ≤ 0.001, ***p ≤ 0.001, ****p ≤ 0.0001, ns = not significant by paired 2-Way ANOVA and a Fisher's LSD test for 5 biological replicates for sparse vs. dense. (**C**) ATP quantitation in cells cultured at indicated densities. Bars = SEM for 3 biological replicates. (**D**) ROS staining in sparse and dense mock- and ketoconazole-treated TS543 cells. Cells were treated with 5 μM ketoconazole for 24 hrs. Sparse = 15,625 cells/cm^2^; Dense = 93,750 cells/cm^2^. (**E**) Quantitation of ROS intensity of cells in D. Circle = ROS fluorescence intensity of a single cell. Bars = SD for at least 250 measured cells, ns = not significant; ****p < 0.0001, Mann-Whitney Test for mock vs. treated. Data shown are representative of 2 biological replicates.

If dense glioma TS cells are preferentially consuming oxygen for cholesterol synthesis, the TCA cycle and electron transport chain might be less active. We observed that four different glioma TS lines (TS543, TS576, TS600, and GBM1) plated at high densities had significantly decreased reactive oxygen species (ROS) than those at lower densities (Supplementary Figure 5A and 5B), suggestive of a decrease in mitochondrial activity in cells grown at high density. While normal astrocytes also had a statistically significant drop in ROS at high densities, ROS levels in the sparse NHAs were much lower than those in the sparse cancer lines. This decrease in ROS at high density is unlikely to be due to depletion of nutrients from the medium: glioma TS cells grown on a density gradient sharing the same medium had decreasing ROS and mitochondrial membrane potential (MMP) with increasing cell density (Figure 4A). It is also unlikely to be due to variations in cell viability with density, as we did not observe any correlation between ROS levels and cell death (Supplementary Figure 5C). It is also unlikely to be due to increased antioxidant capacity in dense cells: while dense glioma cells exhibit a decrease in reduced glutathione, we do not see a consistent corresponding increase in oxidized glutathione or decrease in NADPH (Supplementary Figure 5D). We measured 566 metabolites from two glioma TS lines (TS543 and TS616) to identify metabolic pathways that might be altered by cell density. Both cell lines skewed toward an increase in metabolites in dense cells relative to sparse (Supplementary Figure 6A). Overall, only two categories of metabolites showed density-dependent differences in the same direction in both cell lines: 1) peptides and 2) energy, which comprises the TCA cycle (Supplementary Figure 6B). Consistent with the decreases in ROS, ATP, and MMP seen in dense cancer cells, the metabolomic profiling revealed greater than 1.25-fold decreases in dense cells in most intermediates in the TCA cycle as well as the alpha-ketoglutarate precursors glutamine and glutamate (Figure 4B and Supplementary Figure 6C). Moreover, ATP levels decreased with density in the glioma TS cells but also in the normal astrocytes (Figure 4C). The astrocytes did not increase cholesterol synthesis at high density, but have decreased levels of the mitochondrial outer membrane protein TOM20 and an increase in the mitophagy-mediator Parkin, suggesting that decreased ATP in high density NHAs is due to an increase in mitophagy rather than a shift to cholesterol metabolism (Supplementary Figure 6D and 6E). In the glioma TS cells, decreases in ROS at high density could be reversed by treating cells with the cholesterol synthesis inhibitor, ketoconazole (Figure 4D and 4E). These data suggest that the reduction in mitochondrial respiratory activity in the glioma TS cells may be due to a switch in oxygen utilization from oxidative phosphorylation to cholesterol synthesis.

### Glioma cells upregulate aerobic glycolysis and the pentose phosphate pathway at high cell densities

The condensation of two molecules of acetyl-CoA to acetoacetyl-CoA is the first step of the mevalonate pathway, which synthesizes precursors for protein prenylation, ubiquinone, and cholesterol (Figure 1G). Acetyl-CoA can be generated from multiple sources including beta-oxidation of fatty acids, degradation of branched-chain amino acids, breakdown of ketone bodies (ketolysis), and glycolysis-derived pyruvate [41]. To determine which of these pathways may be contributing acetyl-CoA for cholesterol synthesis, we examined our metabolomic profiling data. There was no evidence of increased beta-oxidation in the dense glioma cells: rather than a decrease in fatty acids consistent with fatty acid catabolism, one glioma cell line increased both saturated and unsaturated long chain fatty acids at high density while a second line had no density-dependent change (Supplementary Figure 7A, 7B). There was also no significant change in the levels of the branched-chain amino acids leucine, isoleucine, or valine (Supplementary Figure 7C) or in the ketone body 3-hydroxybutyrate (Figure 4B and Supplementary Figure 7D) in the dense cancer cells, suggesting that acetyl-CoA was not being generated via either branched-chain amino acid degradation or ketolysis.

In contrast, both the TS543 and TS616 cells upregulated glycolysis at high density; therefore, a likely source of acetyl-CoA for cholesterol synthesis is glycolytically-generated pyruvate (Figure 4B and Supplementary Figure 8A). Interestingly, while glycolysis was up at high density, we did not see a corresponding increase in lactate (Supplementary Figure 8B). Total secreted lactate increased in the culture medium when measured levels were not normalized to cell number, consistent with the commonly observed drop in the pH of the culture medium of confluent cells (Supplementary Figure 8B, left). However, when levels were normalized to cell number, we did not see an increase in either intracellular or extracellular lactate in dense astrocytes or glioma TS cells (Supplementary Figure 8B, middle and right). Overall, these data are consistent with high cell density leading to an upregulation of aerobic glycolysis, but not in a manner consistent with the classically defined “Warburg effect” as there was no ensuing increase in lactate (Figure 4B and Supplementary Figure 8B). More likely, pyruvate is used to make acetyl-CoA, which exits the mitochondria to be used for the mevalonate pathway and cholesterol synthesis.

NADPH is also required to make cholesterol. This metabolite can be generated from multiple pathways, both cytosolic and mitochondrial. The largest source of NADPH in mammalian cells is the cytosolic pentose phosphate pathway and a second important source is serine-driven one-carbon metabolism, in which methylene tetrahydrofolate is oxidized to 10-formyl-tetrahydrofolate in either the cytosol or mitochondria [42]. Because we see an increase in pentose phosphate pathway intermediates in our densely-plated glioma tumor initiating cells, this pathway is likely to be the major source of NADPH generation in our cells (Figure 4B and Supplementary Figure 8C). However, as 10-formyltetrahydrofolate levels were not measured, we cannot rule this out as an additional source of NADPH for cholesterol synthesis in glioma TS cells.

### Disabled cell cycle control leads to dysregulated cholesterol synthesis

**Figure 5:**
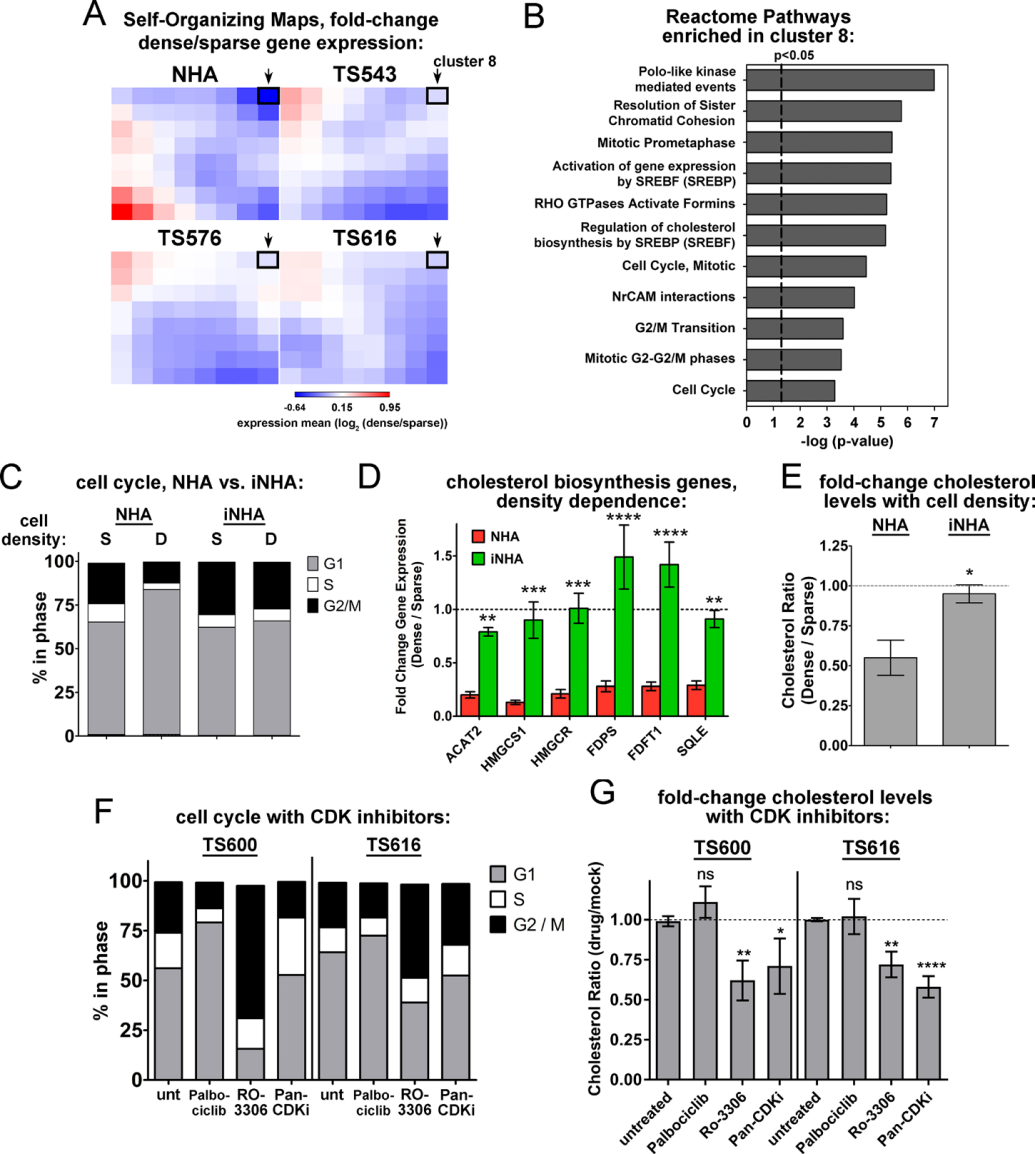
Disabled cell cycle control correlates with constitutive cholesterol synthesis in glioma cells (**A**) Self-Organizing Maps of density-dependent gene expression in NHAs and glioma TS cells. The value of each cluster is the mean of the dense-to-sparse gene expression ratios of each gene set. Cluster 8 is outlined with a black box and indicated with an arrow. (**B**) Reactome pathways enriched in the cluster 8 gene set. All are significant at both *p-value* and FDR *q*-value < 0.05. (**C**) Cell cycle analysis of sparse (S) and dense (D) NHA and iNHA cells. S = 15,625 cells/cm^2^; D = 93,750 cells/cm^2^. Shown is the average of 3 biological replicates. (**D**) Relative gene expression for select enzymes in the mevalonate and cholesterol biosynthesis pathways. Gene expression values derived from quantitative real time PCR normalized to GAPDH and expressed as fold change dense/sparse are shown. Values are the mean of at least 3 biological replicates. **p < 0.005, ***p < 0005, ****p < 0.0001 by 2-way ANOVA with uncorrected Fisher's LSD for NHA vs. iNHA. (**E**) Cholesterol levels in NHA and iNHA as a function of cell density. Cholesterol levels are shown as the ratio of dense to sparse. Bars = SEM for 3 biological replicates, *p < 0.05 with 2-tailed *t-test*. (**F**) Cell cycle analysis of TS600 and TS616 glioma cells treated with CDK inhibitors. Cells were harvested 20 hours after exposure to DMSO (unt), 5 μM Palbociclib, 0.5 μM pan-CDK inhibitor, or 9 μM RO-3306. The average of 3 biological replicates is shown. (**G**) Fold change in cholesterol levels in glioma TS cells treated with CDK inhibitors as in *F*, expressed as the ratio of treated over mock. Bars = SEM for at least 3 biological replicates. ns = not significant, *p < 0.05, **p < 0.005, ****p < 0.0001 by 2-way ANOVA with uncorrected Fisher's LSD for treated vs. untreated.

Figure 1A shows that unlike normal astrocytes, the glioma TS cells do not undergo cell cycle arrest at high density. Consistent with this observation, cell cycle regulation was a second category of pathways enriched by cell density in the REACTOME pathway analysis of the normal astrocytes but not the glioma TS cells. For example, “Mitotic Prometaphase”, “Polo-like kinase mediated events,” and “cell cycle” were all cell density-regulated at a significance of *P* < 0.05 (Figure 1F). We asked whether constitutive cholesterol synthesis in the glioma cells might be related to their disabled cell cycle control. When we clustered density-dependent gene expression changes into self-organizing maps, the cluster that was the most down-regulated in dense normal astrocytes compared to the glioma TS lines (cluster 8, highlighted in black, Figure 5A) contained gene sets for both cell cycle control and cholesterol synthesis, suggesting that they might be coordinately regulated (Figure 5B and Supplementary Table 1). To determine the relationship between the cell cycle and cholesterol synthesis, we used an immortalized normal human astrocyte cell line (iNHA) that expresses E6, E7, and hTERT [43]. Like the glioma TS cells, these cells do not undergo cell cycle arrest at high density, presumably due to p53 and Rb inactivation by E6 and E7 (Figure 5C). Also like the glioma TS cells, the iNHAs fail to turn off expression of genes in the cholesterol synthesis pathway at high density (Figure 5D) and do not decrease cholesterol levels like primary NHAs (Figure 5E).

We next asked whether cell cycle inhibition leads to decreased cholesterol or if decreased cholesterol synthesis causes cell cycle arrest. Inhibition of the cholesterol synthesis pathway with either ketoconazole or Ro 48-8071 did not affect cell cycle dynamics in the glioma TS cells (Supplementary Figure 9A, 9B), though the cells could not undergo lengthy treatments due to toxicity (see below). However, the G2 cell cycle inhibitor, RO-3306, and a pan-CDK inhibitor both decreased cholesterol levels in these cells, while the G1 inhibitor, palbociclib did not (Figure 5F, 5G). Moreover, when cycling primary NHAs were treated with these drugs, the pan-CDK inhibitor and the G2 inhibitor RO-3306 significantly reduced the expression of multiple genes in the cholesterol synthesis pathway, while the G1 inhibitor palbociclib had less of an effect (Supplementary Figure 9C), suggesting that the control of mevalonate and cholesterol synthesis gene expression is largely consigned to G2. Together, these data suggest that disabled cell cycle checkpoint control in the glioma TS cells, most likely in G2 phase, is associated with the constitutive synthesis of cholesterol in dense cells.

### Glioma cells, but not astrocytes, are sensitive to cholesterol synthesis inhibition

**Figure 6:**
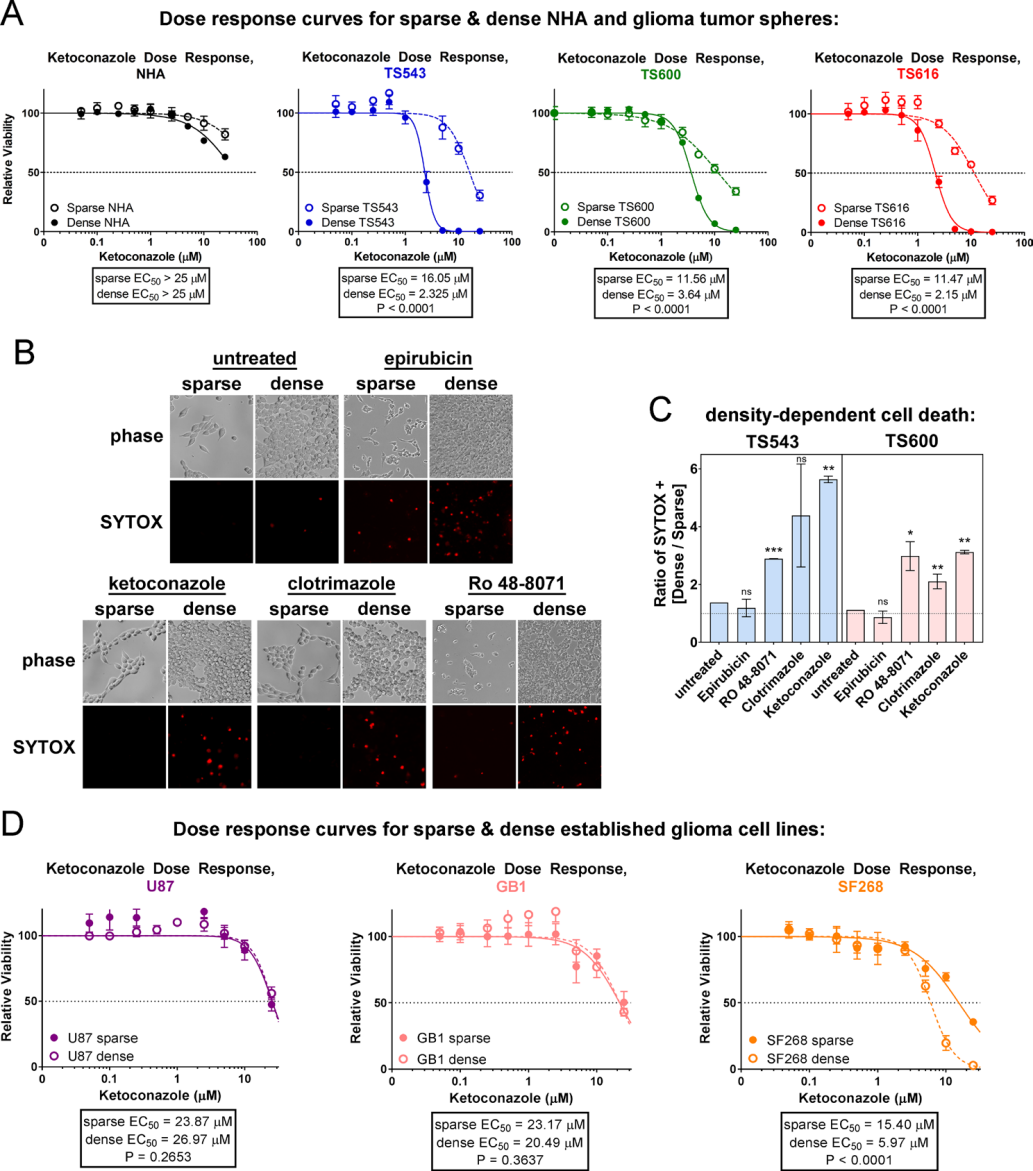
Glioma tumor initiating cells are sensitive to cholesterol synthesis inhibitors (**A**) Ketoconazole dose response curves of sparse and dense NHA and glioma TS cells. Cells were treated for 72 hrs prior to cell viability measurements. Bars = SD. Data shown are representative of at least 3 biological replicates. (**B**) Cell death in TS543 glioma cells. Dead cells were visualized with SYTOX Orange 24 hours after treatment with DMSO (mock), 1 μM epirubicin, 10 μM clotrimazole, 5 μM ketoconazole, or 1 μM Ro 48-8071. Sparse = 15,625 cells/cm^2^; dense = 93,750 cells/cm^2^. (**C**) Quantitation of SYTOX-positive TS543 cells in B and TS600 cells in S9*C*, expressed as the ratio of dense over sparse. Bars = SEM for at least 3 biological replicates. ns = not significant, *p < 0.1, ** *p* < 0.05, ***p < 0.001 by one-sample *t-test* vs. a theoretical mean of one. (**D**) Ketoconazole does response curves of sparse and dense established glioma cell lines. Cells were treated for 72 hrs prior to cell viability measurements. Bars = SD for at least 3 biological replicates.

Constitutive cholesterol synthesis might simply be a consequence of disabled cell cycle control, or it might confer a growth advantage on cancer cells that is selected for in oncogenic transformation. To distinguish between these possibilities, we determined whether glioma TS cells die upon pharmacological inhibition of these pathways. Statins are commonly used cholesterol lowering drugs that inhibit HMGCR in the mevalonate pathway (see Figure 1G), but two different statins, lovastatin and simvastatin, were more toxic to the NHAs than the glioma TS cells, most likely due to their inhibition of not only cholesterol synthesis, but also ubiquinone and dolichol synthesis and prenylation of proteins such as RAS [44] (Supplementary Figure 10A and see Figure 1G). To more selectively target cholesterol synthesis, we treated cells with ketoconazole, which targets CYP51A1 downstream of the mevalonate pathway (see Supplementary Figure 2). We observed that the glioma TS cells were sensitive in the low μM range while the normal astrocytes were resistant (Figure 6A and Supplementary Figure 10B). In addition, densely plated glioma cells were more sensitive to CYP51A1 inhibition than sparse, consistent with our data showing that this pathway is dysregulated in dense cancer cells. We compared density-dependent cell killing between three different inhibitors of the cholesterol synthesis pathway, ketoconazole, clotrimazole (an additional CYP51A1 inhibitor), and Ro 48-8071, to epirubicin, a cytotoxic DNA intercalator. All three of the cholesterol synthesis inhibitors preferentially killed dense glioma cells, while epirubicin was not selective for cell density (Figure 6B, 6C and Supplementary Figure 10C). This is unlikely to be due to differential medium conditions in sparse and dense cultures as cells plated on cell density gradients were also differentially sensitive to cholesterol synthesis inhibitors but not epirubicin (Supplementary Figure 10D). Interestingly, we see that 2 of 3 established, non-stem glioma cell lines tested do not have a density dependent response to ketoconazole, though these lines were relatively insensitive to this drug overall (EC50 > 20 uM, Figure 6D). This could be for multiple reasons: it may be that only cells with stem-like properties are dependent on cholesterol synthesis for viability. Alternatively, because fetal bovine serum provides exogenous cholesterol (> 30 mg/dL in FBS compared to 0 mg/dL in glioma stem cell growth medium [45]), it may be that over the course of the establishment of these lines, the aberrant activation of the signaling pathways required to synthesize cholesterol was lost since they could readily obtain this critical nutrient from the growth medium and therefore had no selection pressure to synthesize it. These results suggest that the cholesterol synthesis pathway is necessary for the survival of high density glioblastoma tumor initiating cells and therefore might be an efficacious therapeutic target in patient tumors.

## DISCUSSION

In an effort to characterize density-dependent differences between normal astrocytes and patient-derived glioblastoma cells, we found that tumor cells have dysregulated the normal controls on cholesterol synthesis. Tumor cells plated at high density increase oxygen consumption and keep cholesterol synthesis gene expression on in order to maintain cholesterol levels, while normal astrocytes maintain consistent oxygen consumption at all densities, but turn off genes in the mevalonate pathway and reduce cholesterol when dense. Relative to sparse cancer cells, dense cancer cells have lower ROS, mitochondrial membrane potential, ATP, and TCA cycle intermediates, and thus are likely increasing oxygen consumption for cholesterol synthesis rather than oxidative phosphorylation. Increased expression of genes in both the mevalonate pathway and downstream cholesterol synthesis pathways correlated with poor prognosis in GBM patients in the TCGA data set, and dense glioma cells, but not astrocytes, were sensitive to cholesterol synthesis inhibitors, suggesting that increased cholesterol synthesis might be a growth advantage for tumor cells. Together, these data suggest that cholesterol synthesis inhibition might be an important strategy for treating GBM.

Loss of cell cycle control through inactivation of the tumor suppressors p53 and RB is a universal hallmark of cancer. Interestingly, we found that the upstream control of cholesterol synthesis relies on intact tumor suppressor regulation of the cell cycle: at high plating densities, both glioma cells and astrocytes engineered to lose p53 and RB failed to undergo contact inhibition of proliferation and to inhibit cholesterol synthesis. In contrast, cholesterol levels in the tumors cells could be decreased by inhibiting the cell cycle with CDK inhibitors. The activities of both p53 and mTOR are known to be affected by cell density [46–48]: because the mTOR pathway is a critical regulator of cell metabolism that is regulated by p53 [49], one possible mechanism by which high cell density inhibits cholesterol synthesis in normal cells but not cancer cells may be through p53-dependent inhibition of mTOR. As we have limited capabilities to therapeutically target tumor suppressor loss in cancer, the inhibition of cholesterol synthesis might be an important clinical alternative.

Cholesterol is critical for multiple cellular processes, including membrane integrity and lipid raft and caveolae formation, yet its role in cancer is not well-defined. It is a precursor for steroid hormones, bile acids, and Vitamin D, and oxygenated cholesterol modulates the activity of multiple nuclear hormone receptors including LXRA, ESR, and ESRRA [14–16]. Post-translational modification through direct cholesterol binding can also regulate the activity of Hedgehog, an important developmental signaling regulator that drives the growth of basal cell carcinomas and medulloblastomas [50, 51]. Moreover, quantitative profiling of HeLa cell proteins by mass spectrometry revealed that cholesterol is bound to more than 250 proteins in HeLa cells, including sterol biosynthetic enzymes, glycerophospholipid metabolic enzymes, protein glycosylation and degradation pathways, and protein networks that regulate membrane structure and dynamics [52]. It may be that an under-appreciated role of tumor suppressors is the regulation of these cholesterol-dependent signaling pathways.

Acetyl-CoA is required for cholesterol synthesis, and we observed that dense cells may upregulate glycolysis in order to generate pyruvate for acetyl-CoA synthesis. Cancer metabolism studies have historically focused on this metabolic pathway due to early observations by Otto Warburg that tumor cells have increased glucose uptake, which may fuel energy production through aerobic glycolysis followed by lactic acid fermentation in the cytosol [53]. In dense glioma TS cells, we saw increased glycolysis and decreased TCA cycle, consistent with a “Warburg Effect”, but without an accompanying increase in lactate. We propose that the glioma TS cells may increase aerobic glycolysis at high density in order to generate acetyl-CoA for the mevalonate pathway and cholesterol synthesis, resulting in a downregulation of the TCA cycle and lowering of cellular ATP levels. Recent *in vivo* metabolic studies showed that brain tumors can use acetate as an alternative to glucose for acetyl-CoA generation [54–56]. Perhaps acetate-derived acetyl-CoA can also be used for the mevalonate pathway and cholesterol synthesis in GBM.

The unique metabolic requirements of the brain might make glioblastoma particularly suitable for cholesterol pathway targeting. Cholesterol is synthesized *de novo* in the brain because the blood-brain barrier prevents the uptake of serum cholesterol. In the mouse, cholesterol synthesis in the brain is high until the second week after birth, then decreases and remains low in adults [57]. Consequently, inhibition of cholesterol synthesis might have minimal impact on normal cells in the adult brain because these pathways have lower activity, and our data show that normal astrocytes are less sensitive to cholesterol synthesis inhibitors than cancer cells. This strategy might also elicit minimal side effects because all other tissues can utilize dietary cholesterol as an alternative to *de novo* synthesis. Previous work showed that statins, which inhibit the mevalonate pathway, are toxic to brain tumor cells [58], but we found that inhibiting this pathway might also be toxic to normal astrocytes, possibly through other branches of the mevalonate pathway, such as the isoprenylation of proteins including Ras and Rho family members [44]. Our work suggests that a further refinement of this strategy is to specifically inhibit one downstream branch of the mevalonate pathway, cholesterol synthesis. CYP51A1 is an ER resident cytochrome P450 required for cholesterol biosynthesis. Multiple CYP51 inhibitors are FDA-approved and safely used for systemic fungal infections, such as ketoconazole, itraconazole, voriconazole, and fluconazole. Interestingly, while this manuscript was under revision, another group found that activation of the Liver X Receptor via the LXR agonist, LXR-623, is also lethal to glioma cells in cell culture and in orthotopic animal models, presumably via lowering cellular cholesterol levels through the activation of the cholesterol efflux pump, ABCA1 [59]. Therefore, cholesterol metabolism offers multiple points of intervention to be explored for therapeutic targeting.

Finally, glioblastoma is largely driven by RTK-PI3K signaling [5], but targeted therapies against effectors of this pathway have been ineffective, most likely for multiple reasons including pathway redundancy [9] and cell-to-cell tumor heterogeneity [10, 11]. We observed constitutive cholesterol synthesis in multiple glioma tumor sphere lines harboring a variety of alterations in this pathway, including both EGFR and PDGFRA amplification and PTEN deletion, suggesting that cholesterol synthesis inhibition might be useful across a wide genomic spectrum.

## MATERIALS AND METHODS

### Cells and cell culture

See Supplemental Text for cell sources and culture conditions. Glioma TS cells were cultured as spheroids on uncoated plates or adherent on Poly-L-ornithine (1.5 mg/mL, Sigma) and laminin coated plates (10 μg/mL, Life Technologies). Unless otherwise stated, sparse cells were cultured at 15,625 cells/cm^2^ on laminin and at 50,000 cells/mL in suspension and dense cells were cultured at 93,750 cells/cm^2^ on laminin and at 300,000 cells/mL in suspension. For cell density gradients, a four-well chamberslide was placed in a 50 mL reagent reservoir (Corning), which provided a solid 55° angled surface. 2 × 10^5^ cells in 750 μL were pipetted into the bottom of each well and the reservoir and slide transferred into an incubator overnight. Slides were then laid flat for 6–24 hours prior to analysis.

### Statistical analyses

All statistical analyses were performed with GraphPad Prism 7.

### Cell cycle analysis and inhibition

Cell cycle profiles were generated using flow cytometry. Cells were plated sparse (15,625 cells/cm^2^) or dense (93,750 cells/cm^2^) for 24–48 prior to harvest. 1×10^6^ cells were resuspended in 200 μL PBS and fixed by dropwise addition of 500 μL ice cold 100% ethanol while vortexing. Fixed cells were stored overnight at −20°C. The following day, cells were centrifuged at 200 × g for 5 minutes and PBS/ethanol aspirated. Cells were stained with 10 μg/mL propidium iodide in PBS containing 100 ug/mL RNase for 30 minutes prior to flow cytometry. 10,000 events were quantitated. For cell cycle inhibition, cells were treated with 5 μM Palbociclib (LC Laboratories), 0.5 μM Pan-CDK inhibitor (Calbiochem) or 9 μM RO-3306 (Sigma) for 20 hours prior to fixing.

### Gene expression profiling and quantitative PCR

NHAs were grown as adherent cultures and glioma TS cells were grown as spheroids for 4 days prior to harvesting for RNA using the RNeasy Mini Kit (Qiagen). RNA purity and quality was quantitated using an Agilent 2100 Bioanalyzer with the RNA 6000 Nano Kit (Agilent). RNA was only used for microarrays if it had 260/280 ratios of 1.7–2.1, 28S/18 S ratios around 2, and an RNA integrity number of at least 7. Gene expression profiling was performed on PrimeView Microarrays using the GeneChip 3*′* IVT Express Labeling Kit (Affymetrix). Microarrays were stained and washed on a GeneChip Fluidics Station 450 and scanned on a GeneChip Scanner (Affymetrix). See SI Text for microarray processing and data analysis and for quantitative PCR. Gene expression profiling data can be found in the GEO repository at http://www.ncbi.nlm.nih.gov/geo, Accession Number GSE79097.

### Survival analyses

Survival analyses for gene expression in TCGA patient samples were generated with the Glioblastoma BioDiscovery Portal [33] (http://gbm-biodp.nci.nih.gov/) with the 173 patients in the “Verhaak 840 Core” group of the TCGA dataset. The gene expression values for gene sets from each pathway were condensed into a single value for each tumor, termed a metagene score, by averaging the expressions of the individual genes. The samples were ordered according to the metagene score. Any genes in the pathways that are not listed were absent from the arrays used in profiling the genes for the survival curves. Patients were stratified into 4 quartiles, and comparisons were made between the lowest quartile (< = 25%) vs. the highest quartile (> = 75%), using the following settings: Participants = Verhaak core, Experiments = 3-Platform Aggregates, stratification according to increasing Prognostic Index Levels by 1 Qt vs 4 Qt, and Cox model covariates Age and MGMT methylation status.

### Pathway enrichment

Pathway analysis was performed using GSEA v2.2.2 (Broad Institute). CEL files of at least 3 replicates for each condition were analyzed using the following settings: 1000 permutations, permutation type = gene set, enrichment statistic = weighted, metric for ranking genes = log2 ratio of classes, collapsing mode for genes with multiple probe sets = max probe. For REACTOME and PANTHER pathway analyses, a list of genes downregulated in the dense NHAs more than 1.5-fold relative to sparse at a *p-value* of < 0.05 was generated from the microarray data processed in Partek (see Supplementary Methods for details.) Differentially regulated pathways were found for this gene list with REACTOME [32] at http://www.reactome.org/ and PANTHER [31] at http://pantherdb.org/.

### Cholesterol measurements and inhibition

See Supplemental Text for details of cholesterol extraction. Ketoconazole and clotrimazole were obtained from the National Cancer Institute Developmental Therapeutics Program and Ro 48-8071 was from MedChem Express. Lovastatin and Simvastatin were obtained from Cayman Chemical.

### Metabolic measurements

See Supplemental Text.

### Cell death assays

### Dose response curves

Cells were plated as spheroids or on poly-L-ornithine/laminin on white 96-well plates in 75 μl of medium. The following day, cells were drugged via addition of 25 μl of 4× drug dilution and incubated for 72 hrs. Images of cells were captured on an EVOS FL fluorescence microscope (ThermoFisher) prior to harvesting with CellTiter-Glo (Promega). Luminescence analyzed on a Wallac Victor2 1420 Multilabel Counter.

### Cell death staining

Cells were incubated in 1μM SYTOX Orange (Life Technologies) and 2 μg/mL Hoechst 33342 for 30 minutes. Stain medium was replaced with fresh, warm media and cells immediately imaged. To quantitate death in wells of defined density, ImageJ was used to count total nuclei (Hoechst) and SYTOX-positive nuclei to calculate the percentage of dead cells per well. Three to ten wells were counted to reach a minimum of 1500 cells per condition.

### Statistical analysis of multiwell plates

Outlier analysis was performed on all 96-well plates using the method of the Iglewicz and Hoaglin described in the NIST Engineering Statistics Handbook Section 1.3.5.17 (http://www.itl.nist.gov/div898/handbook/eda/section3/eda35h.htm), using a modified Z-score cutoff of 3.5.

## SUPPLEMENTARY MATERIALS FIGURES AND TABLES




