# Supplementary material for: Disabled cell density sensing leads to dysregulated cholesterol synthesis in glioblastoma
Source: Oncotarget. 2017 Jan 19;8(9):14860–75. doi: 10.18632/oncotarget.14740 (PMC5362450; doi:10.18632/oncotarget.14740)
Supplement: Supplementary file 1 [file oncotarget-08-14860-s001.pdf]

Disabled cell density sensing leads to dysregulated cholesterol synthesis in glioblastoma

Supplementary Materials

SUPPLEMENTAL INFORMATION

Supplemental Text

Cells and cell culture

All cells were cultured at 37°C and 5% CO_2. The E6/E7/hTERT immortalized normal human astrocytes were provided by Russell Pieper (UCSF) and cultured in DMEM +10% heat inactivated FBS [1]. Normal human astrocytes (NHA) were purchased from ScienCell Research Laboratories, cultured in Astrocyte Medium (ScienCell Research Laboratories), and passaged by trypsinization with TrypLE Express (Life Technologies). NHAs were used until passage 12. Glioma TS cells were obtained from Cameron Brennan (MSKCC) and derived from surgically resected patient tumors [2, 3]. Glioma TS cells were cultured in DMEM:F12 (HyClone) with 1× B-27 Supplement without Vitamin A (Life Technologies), 10 ng/mL EGF (Peprotech), 10 ng/mL bFGF (R&D Systems), Primocin (InvivoGen), and 2 ug/mL heparin (Sigma). U87 and GB1 established glioma cells were obtained from Lynda Chin (MDACC), and SF268 established glioma cells were obtained from the NCI-Frederick DCTD Cancer Cell Line Repository and were cultured in DMEM with 10% heat inactivated FBS and Normocin (InvivoGen). WS1 and CCD-18Co cells were obtained from ATCC and cultured in MEM (Life Technologies), 15% heat inactivated FBS (HyClone), 2× NEAA (Life Technologies), 2× vitamins (Life Technologies), Primocin (InvivoGen) and 1:2000 Gibco β-mercaptoethanol (Life Techologies #21985-023). HT29, HCT116, NCI-H3255, NCI-H522 cells were obtained from Ron DePinho (MDACC) and were cultured in RPMI with 10% heat inactivated FBS and Normocin (InvivoGen).

Quantitative real-time PCR

Glioma TS cells and NHAs were grown as adherent cultures on Poly-L-Ornithine/Laminin coated plates in glioma TS cell medium for 24 hours (NHA), 48 hours (TS543, TS616), or 72 hours (TS576) prior to harvesting with the RNAeasy Mini Kit (Qiagen). For quantitative PCR, cDNA was generated using the iScript Advance cDNA Synthesis Kit for RT (Bio-Rad), and PCR was performed using KAPA SYBR FAST qPCR kits (KAPA Biosystems) on an Applied Biosystems 7500 Real Time PCR System. Fold-change gene expression for dense cells relative to sparse was calculated by normalizing to GAPDH followed by the Comparative C_T Method. Primer sequences:

ACAT2: sense: TCAATGAAGCCTTTGCAG, anti: CAATATTGACCTTCTCTGGG - Sigma KiCqStart Primers

HMGCS1: sense: TTGGCTTCATGATCTTTCAC, anti: AATTTAACATCCCCAAAGGC - Sigma KiCqStart Primers

HMGCR: sense: ACTTCGTGTTCATGACTTTC, anti: GACATAATCATCTTGACCCTC - Sigma KiCqStart Primers

FDPS: sense: GTCAGATTCACTGAAAAGAGG, anti: GTCAAGGTAATCATCCTGAATC - Sigma KiCqStart Primers

FDFT1: sense: ACGATCTCCCTTGAGTTTAG, anti: TGCTTATCCAAAAACTCTGCAATC - Sigma KiCqStart Primers

SQLE: sense: GATCACCTGAAAGAACCATTC, anti: TTCATCGATGAAGGAGGAAGAATC - Sigma KiCqStart Primers

INSIG1: sense: ACCCCACAAATTTAAGAGAG, anti: TTCTGGAACGATCAAATGTC, Sigma KiCqStart Primers

SREBF1: sense: CAGCGAGTCTGCCTTGATG, anti: GCCCCTGTAACGACCACTG, from Primer Bank[4] (https://pga.mgh.harvard.edu/primerbank/)

SREBF2: sense: CAGCAGGTCAATCATAAACTG, anti: GGACATTCTGATTAAAGTCCTC - Sigma KiCqStart Primers

ABCA1: sense: TCTCACCACTTCGGTCTCCA, anti: TTCCTCGCCAAACCAGTAGG, from Primer-BLAST (http://www.ncbi.nlm.nih.gov/tools/primer-blast/)

APOE: sense: AGGAAGATGAAGGTTCTGTG, anti: CCTTGAGTCCTACTCAGC - Sigma KiCqStart Primers

GAPDH: sense: ACAGTTGCCATGTAGACC, anti: TTTTTGGTTGAGCACAGG - Sigma KiCqStart Primer

Cell culture and RNA harvesting for microarrays

Glioma TS cells

Cells were grown as suspension cultures. 3–4 biological replicates of each cell line were harvested on different days as matched sets of sparse and dense. Sparse = 5×10^4 cells/mL, in 3 Corning T75 flasks (= 75 cm^2); dense = 3×10^5 cell/mL, in 1 Corning T75 flask (= 75 cm^2). Day 1: Cells were plated 7.5 mL medium. Day 2: Cells were fed with 2.5 mL medium. Day 5: cells were harvested with the RNeasy Mini Kit, with modification: RW1 wash = 350 µL RW1, followed by on column DNA digestion with Ambion Turbo DNase (#AM2238) 37°C 30 minutes (50 µL total), then remaining 350 µL RW1 wash. *NHAs:* 3 biological replicates were harvested on different days as matched sets of sparse and dense. Sparse = 3.5×10^5 cells and dense = 5.2×10^6 cells on 10 mm Corning TC dishes (= 55 cm^2). Cells were plated on day 1 in 12 mL medium, and then fed with 4 mL more on day 2. Final conc. of cells = 5.3×10^4/mL sparse, 3.25×10^5/mL dense. Cells were harvested on Day 5 as for glioma cells. RNA purity and quality was quantitated using an Agilent 2100 Bioanalyzer with the RNA 6000 Nano Kit (Agilent). RNA was only used for microarrays if it had 260/280 ratios of 1.7–2.1, 28S/18S ratios around 2, and an RNA integrity number of at least 7.

Microarray processing and data analysis

Microarrays were processed using Partek Genomics Suite version 6.6. Normalization was performed using RMA background correction with the following Partek settings: adjust for GC content, quantile normalization, probeset summarization = mean. Batch removal was performed across experimental replicates. Probesets were collapsed to gene IDs from the probeset with the lowest p-value. Cell density enriched genes were found via two-sample *t*-test, equal variance comparing sparse and dense for each cell line. Hierarchical clustering analysis was performed on processed data in Partek Genomics Suite using the following parameters: columns were shifted to mean of zero, row and column dissimilarity = Euclidean, row and column method = average linkage. Venn diagrams were generated in Partek Genomics Suite. Self-Organizing Maps were generated for an 8x8 grid using 20,000 training iterations.

Survival and gene expression analyses

The Kaplan Meier method and log-rank test was used on GBM TCGA data to assess the clinical relevance of genes in the cholesterol and mevalonate pathways. For each gene, comparisons between patients with low and high expression were made by separating patients into quantile groups and selecting either low expression group (gene expression value < = 2nd quantile) and high expression group (gene expression value > = 4th quantile). Survival analysis was performed in R using "survival" package and Log-rank p-value computed for each plot. Z-scores for gene expression relative to normal brain were downloaded from cBioPortal (http://www.cbioportal.org/), up-to-date as of December 2016.

Cholesterol extraction and quantitation

Cholesterol was extracted using a modified Bligh and Dyer method carried out entirely with glass labware [5]. Briefly, 0.5–1 × 10^6 live cells were resuspended in 200 µL PBS and either frozen on dry ice or immediately processed. To cell suspension, 700 µL of a 2:1 methanol:chloroform solution was added and the tube vortexed followed by addition of 300 µL chloroform and a second round of vigorous vortexing. Finally, 250 µL of 1 M NaCl was added and mixed by vortexing. Samples were centrifuged at 3000 × g for 15 minutes at 4°C and the organic (bottom) phase collected with a Pasteur pipette into a fresh glass test tube. Samples were either vacuum dried for 1 hour or allowed to air dry in a chemical hood for no more than 18 hours. Cholesterol was resuspended in 80 µL of 5X Amplex buffer with periodic vortexing for 1 hour, followed by dilution with additional 320 µL water (to final 1X buffer concentration). Amplex Red Cholesterol Assay (Life Technologies) was carried out according to manufacturer instructions. To analyze cholesterol levels by flow cytometry, 0.5–1.0 × 10^6 cells were fixed in 1% paraformaldehyde for 25 minutes, centrifuged 10 minutes at 1000 rpm, and resuspended in 1.5 µg/mL Filipin III (Sigma #F4767). Cells were stained for 2 hours then analyzed using an LSR Fortessa flow cytometer (BD Biosciences). 10,000 ungated events were analyzed with FlowJo Single Cell Analysis software.

Metabolic measurements

Oxygen consumption rates (OCR) were evaluated using an XFe96 Extracellular Flux Analyzer (Seahorse Bioscience). XFe96 plates were coated with 0.01 ug/mL poly-L-ornithine overnight, washed 3x with water, then incubated with 10 µg/mL laminin in glioma TS cell medium overnight. Wells were rinsed 1x with medium and cells seeded at indicated densities for 24 hours. The XFe96 sensor cartridge was hydrated overnight in XF calibrant, per manufacturer instructions. Prior to analysis, cells were rinsed twice with XF assay medium (XF Base Medium supplemented with 17.5 mM glucose, 0.5 mM pyruvate, 2.5 mM glutamine, 10 ng/mL EGF, 10 ng/mL bFGF, and 2 µg/mL heparin) then incubated for one hour in XF assay medium at 37°C in a CO_2-free incubator. Injection cartridges were loaded with compounds as indicated. For

each experiment, average OCR values were determined from a minimum of 6 technical replicates and a minimum of 3 measurement cycles per condition. To account for cell density, all extracellular flux assays were normalized to SYTO 60 Red Fluorescent Nucleic Acid Stain (Life Technologies) after completion of the experiment. Briefly, media was removed from the cell plate and replaced with 1:5000 SYTO 60 in TBS. Cell plate was incubated with SYTO 60 at room temperature for 30 minutes, then rinsed with TBS. After removing all liquid from the wells, the plate was scanned using a LI-COR Odyssey CLx. Well intensities were quantitated using Image Studio software (LI-COR).

ROS, MMP, and measurements

MitoTracker Red CMXRos (Life Technologies) was used at a concentration of 100 nM to assess mitochondrial membrane potential (MMP). CellROX Green (Life Technologies) was used at a concentration of 2.5 μM to assess cellular ROS. Both were diluted as indicated in pre-warmed (37°C) medium and the slide incubated with the solution for 15–30 minutes. Stain was then replaced with fresh, warmed medium and slides imaged. Images were captured on an EVOS fl fluorescence microscope (ThermoFisher). ROS quantitation was performed using ImageJ. Five random fields were imaged for each well. For each field, the background was calculated as an average of four cell-free areas, and then the signal intensity of fifty random, representative cells was measured. For each cell, the Corrected Fluorescence = Integrated Density – (Area * Mean Background Fluorescence). Dot plots depict these 250 data points and mean and standard deviation were calculated in GraphPad Prism. Each set of images within a figure panel were cropped and levels were adjusted using identical settings in Adobe Photoshop. For ATP measurements, cells were plated at the indicated densities in 75 μL media per well of a white 96-well plate. After 24 hours, 75 μL CellTiter-Glo (Promega) was added per well and plates mixed for 2 minutes at 450 rpm. Plates were incubated in the dark for 15–20 minutes then the luminescence analyzed on a Wallac Victor2 1420 Multilabel Counter. For each experiment, the average luminescence was calculated from four technical replicates per density. Relative ATP level was calculated by dividing the average luminescence for each density by the average luminescence of the samples plated at 15,625 cells/cm^2 ("sparse"). The ratios of 3 independent biological replicates were averaged and presented as mean +/− standard error of the mean.

Lactate measurements

Analysis of lactate levels was performed using the L-Lactate Assay Kit (Cayman Chemical Company) according to manufacturer protocols for intra- and extracellular L-lactate. Prior to analysis, cells were plated sparsely or densely for 24 hours then collected and counted. Equal cell numbers were processed for analysis of intracellular lactate in sparse and dense samples. To analyze extracellular lactate, 600 μL media supernatant was collected after cells were pelleted for counting. Samples were process as indicated in manufacturer's protocol and analyzed. To account for differential cell density, raw fluorescence values were normalized by multiplying by a density factor derived from the number of cells in the sparse and dense cultures.

Metabolomics

TS543 and TS616 cells were cultured as spheroids at 50,000 cells/mL (sparse) or 300,000 cells/mL (dense) in suspension. 1×10^7 cells were pelleted and flash frozen in liquid nitrogen. Metabolites were profiled by Metabolon (Durham, NC, USA) as follows: following normalization to Bradford protein concentration, log transformation and imputation of missing values, if any, with the minimum observed value for each compound, *Sample Accessioning:* Following receipt, samples were inventoried and immediately stored at –80°C. Each sample received was accessioned into the Metabolon LIMS system and was assigned by the LIMS a unique identifier that was associated with the original source identifier only. This identifier was used to track all sample handling, tasks, results, etc. The samples (and all derived aliquots) were tracked by the LIMS system. All portions of any sample were automatically assigned their own unique identifiers by the LIMS when a new task was created; the relationship of these samples was also tracked. All samples were maintained at −80°C until processed. *Sample Preparation:* Samples were prepared using the automated MicroLab STAR® system from Hamilton Company. A recovery standard was added prior to the first step in the extraction process for QC purposes. To remove protein, dissociate small molecules bound to protein or trapped in the precipitated protein matrix, and to recover chemically diverse metabolites, proteins were precipitated with methanol under vigorous shaking for 2 min (Glen Mills GenoGrinder 2000) followed by centrifugation. The resulting extract was divided into five fractions: one for analysis by UPLC-MS/MS with positive ion mode electrospray ionization, one for analysis by UPLC-MS/MS with negative ion mode electrospray ionization, one for LC polar platform, one for analysis by GC-MS, and one sample was reserved for backup. Samples were placed briefly on a TurboVap (Zymark) to remove the organic solvent. For LC, the samples were stored overnight under nitrogen before preparation for analysis. For GC, each sample was dried under vacuum overnight before preparation for analysis. *QA/QC:* Several types of controls were analyzed in concert with the experimental samples: a pooled matrix sample generated by taking a small volume

of each experimental sample (or alternatively, use of a pool of well-characterized human plasma) served as a technical replicate throughout the data set; extracted water samples served as process blanks; and a cocktail of QC standards that were carefully chosen not to interfere with the measurement of endogenous compounds were spiked into every analyzed sample, allowed instrument performance monitoring and aided chromatographic alignment. Instrument variability was determined by calculating the median relative standard deviation (RSD) for the standards that were added to each sample prior to injection into the mass spectrometers. Overall process variability was determined by calculating the median RSD for all endogenous metabolites (i.e., non-instrument standards) present in 100% of the pooled matrix samples. *Ultrahigh Performance Liquid Chromatography-Tandem Mass Spectroscopy (UPLC-MS/MS):* The LC/MS portion of the platform was based on a Waters ACQUITY ultra-performance liquid chromatography (UPLC) and a Thermo Scientific Q-Exactive high resolution/accurate mass spectrometer interfaced with a heated electrospray ionization (HESI-II) source and Orbitrap mass analyzer operated at 35,000 mass resolution. The sample extract was dried then reconstituted in acidic or basic LC-compatible solvents, each of which contained 8 or more injection standards at fixed concentrations to ensure injection and chromatographic consistency. One aliquot was analyzed using acidic positive ion optimized conditions and the other using basic negative ion optimized conditions in two independent injections using separate dedicated columns (Waters UPLC BEH C 18–2.1 × 100 mm, 1.7 µm). Extracts reconstituted in acidic conditions were gradient eluted from a C18 column using water and methanol containing 0.1% formic acid. The basic extracts were similarly eluted from C18 using methanol and water, however with 6.5 mM Ammonium Bicarbonate. The third aliquot was analyzed via negative ionization following elution from a HILIC column (Waters UPLC BEH Amide 2.1 × 150 mm, 1.7 µm) using a gradient consisting of water and acetonitrile with 10mM Ammonium Formate. The MS analysis alternated between MS and data-dependent MS2 scans using dynamic exclusion, and the scan range was from 80–1000 m/z. Raw data files are archived and extracted as described below. *Gas Chromatography-Mass Spectroscopy (GC-MS):* The samples destined for analysis by GC-MS were dried under vacuum for a minimum of 18 h prior to being derivatized under dried nitrogen using bistrimethyl-silyltrifluoroacetamide. Derivatized samples were separated on a 5% diphenyl / 95% dimethyl polysiloxane fused silica column (20 m × 0.18 mm ID; 0.18 um film thickness) with helium as carrier gas and a temperature ramp from 60° to 340°C in a 17.5 min period. Samples were analyzed on a Thermo-Finnigan Trace DSQ fast-scanning single-quadrupole mass spectrometer using electron impact ionization (EI) and operated at unit mass resolving power. The scan range was from 50–750 m/z. Raw data files are archived and extracted as described below. *LIMS:* The purpose of the Metabolon LIMS system was to enable fully auditable laboratory automation through a secure, easy to use, and highly specialized system. The scope of the Metabolon LIMS system encompasses sample accessioning, sample preparation and instrumental analysis and reporting and advanced data analysis. All of the subsequent software systems are grounded in the LIMS data structures. It has been modified to leverage and interface with the in-house information extraction and data visualization systems, as well as third party instrumentation and data analysis software. *Data Extraction and Compound Identification:* Raw data was extracted, peak-identified and QC processed using Metabolon's hardware and software. These systems are built on a web-service platform utilizing Microsoft's .NET technologies, which run on high-performance application servers and fiber-channel storage arrays in clusters to provide active failover and load-balancing. Compounds were identified by comparison to library entries of purified standards or recurrent unknown entities. Metabolon maintains a library based on authenticated standards that contains the retention time/index (RI), mass to charge ratio (m/z), and chromatographic data (including MS/MS spectral data) on all molecules present in the library. Furthermore, biochemical identifications are based on three criteria: retention index within a narrow RI window of the proposed identification, accurate mass match to the library +/− 0.005 amu, and the MS/MS forward and reverse scores between the experimental data and authentic standards. The MS/MS scores are based on a comparison of the ions present in the experimental spectrum to the ions present in the library spectrum. While there may be similarities between these molecules based on one of these factors, the use of all three data points can be utilized to distinguish and differentiate biochemicals. More than 3300 commercially available purified standard compounds have been acquired and registered into LIMS for distribution to both the LC-MS and GC-MS platforms for determination of their analytical characteristics. Additional mass spectral entries have been created for structurally unnamed biochemicals, which have been identified by virtue of their recurrent nature (both chromatographic and mass spectral). These compounds have the potential to be identified by future acquisition of a matching purified standard or by classical structural analysis. *Curation:* A variety of curation procedures were carried out to ensure that a high quality data set was made available for statistical analysis and data interpretation. The QC and curation processes were designed to ensure accurate and consistent identification of true chemical entities, and to remove those representing system artifacts, misassignments, and background noise. Metabolon data analysts use proprietary visualization and interpretation software to confirm the consistency of peak identification

among the various samples. Library matches for each compound were checked for each sample and corrected if necessary. *Metabolite Quantification and Data Normalization:* Peaks were quantified using area-under-the-curve. For studies spanning multiple days, a data normalization step was performed to correct variation resulting from instrument inter-day tuning differences. Essentially, each compound was corrected in run-day blocks by registering the medians to equal one (1.00) and normalizing each data point proportionately (termed the "block correction"). For studies that did not require more than one day of analysis, no normalization is necessary, other than for purposes of data visualization. In certain instances, biochemical data may have been normalized to an additional factor (e.g., cell counts, total protein as determined by Bradford assay, osmolality, etc.) to account for differences in metabolite levels due to differences in the amount of material present in each sample. Identification of metabolites with a statistically significant fold change in dense cells versus sparse cells was performed in GraphPad Prism 7.01 using a grouped two-way ANOVA analysis (grouped by cell line and cell harvest date), without correction for multiple comparisons using a Fisher's LSD test.

Immunoblots

Cells were plated either sparse (15,625 cells/cm2) or dense (93,750 cells/cm2) on laminin-coated plates for 24 hours prior to lysing in RIPA. 30 µg of whole cell extracts were separated on 4–12% NuPage Bis-Tris gradient gels in MES buffer (Life Technologies), then transferred onto nitrocellulose (Bio-Rad). Membranes were blocked in 5% milk (Cell Signaling #9999) in TBST, then probed with antibodies to TOM20 (Santa Cruz , #SC-17764), Parkin (Cell Signaling, #4211), and HSP90 (GeneTex #GTX109753). Blots were probed with fluorescently-labeled secondary antibodies, and bands were scanned and quantitated on a LI-COR Odyssey CLx using Image Studio (LI-COR).

REFERENCES

1. Sonoda Y, Ozawa T, Hirose Y, Aldape KD, McMahon M, Berger MS, Pieper RO. Formation of intracranial tumors by genetically modified human astrocytes defines four pathways critical in the development of human anaplastic astrocytoma. Cancer Res. 2001; 61:4956–60.
2. Ozawa T, Brennan CW, Wang L, Squatrito M, Sasayama T, Nakada M, Huse JT, Pedraza A, Utsuki S, Yasui Y, Tandon A, Fomchenko EI, Oka H, et al. PDGFRA gene rearrangements are frequent genetic events in PDGFRA-amplified glioblastomas. Genes Dev. 2010; 24:2205–18. doi: 10.1101/gad.1972310.
3. Szerlip NJ, Pedraza A, Chakravarty D, Azim M, McGuire J, Fang Y, Ozawa T, Holland EC, Huse JT, Jhanwar S, Leversha MA, Mikkelsen T, Brennan CW. Intratumoral heterogeneity of receptor tyrosine kinases EGFR and PDGFRA amplification in glioblastoma defines subpopulations with distinct growth factor response. Proc Natl Acad Sci USA. 2012; 109:3041–6. doi: 10.1073/pnas.1114033109.
4. Wang X, Spandidos A, Wang H, Seed B. PrimerBank: a PCR primer database for quantitative gene expression analysis, 2012 update. Nucleic Acids Res. 2012; 40: D1144–9. doi: 10.1093/nar/gkr1013.
5. Bligh EG, Dyer WJ. A rapid method of total lipid extraction and purification. Can J Biochem Physiol. 1959; 37:911–7. doi: 10.1139/o59-099.
6. Bloch K. The biological synthesis of cholesterol. Science. 1965; 150:19–28. doi.
7. Kandutsch AA, Russell AE. Preputial gland tumor sterols. 3. A metabolic pathway from lanosterol to cholesterol. J Biol Chem. 1960; 235:2256–61.

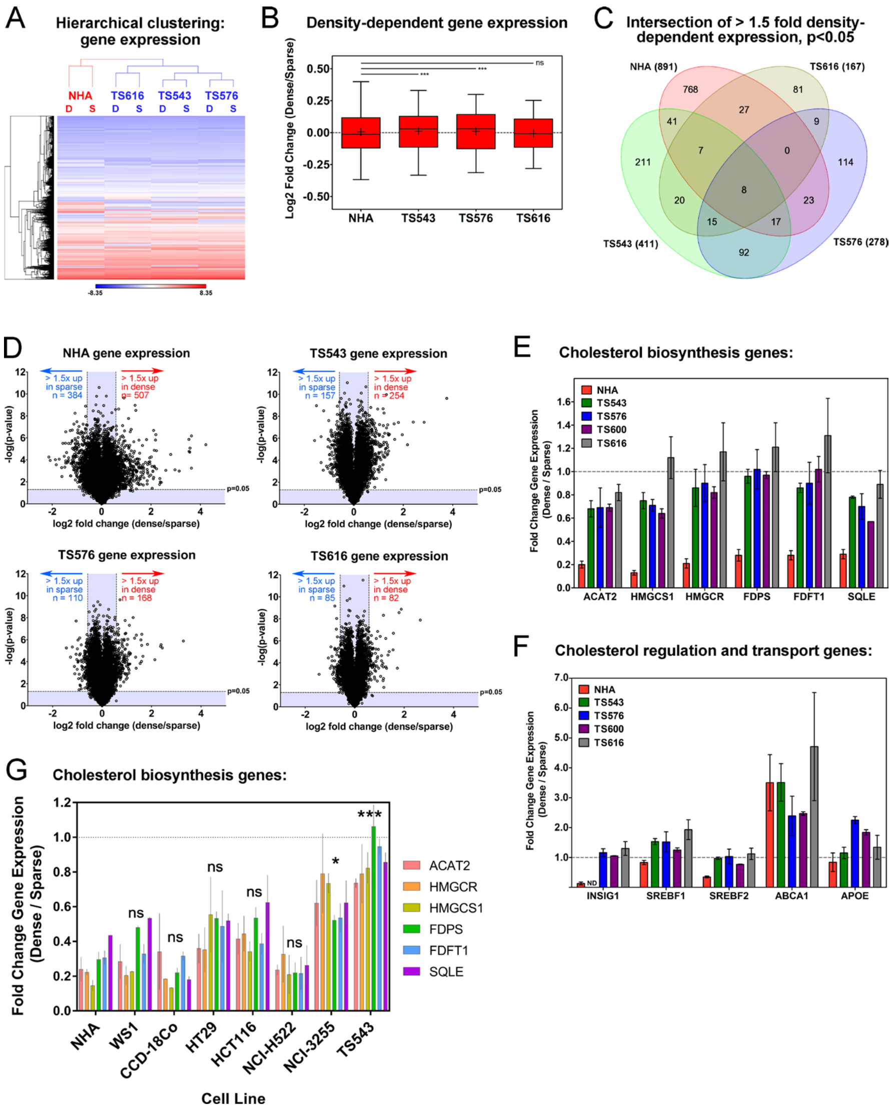

Supplementary Figure 1: Cholesterol biosynthesis pathways are dysregulated in glioma cells plated at high density. (**A**) Hierarchical clustering of NHA and glioma TS cells sparse (S, 15,625 cells/cm^2) and dense (D, 93,750 cells/cm^2) gene expression. Normalized, shifted, compressed gene values are shown (see Supplemental Methods). (**B**) Box and whisker plot of density-dependent gene expression in NHAs and TS lines, shown as $\log_2$ fold change in gene expression, dense over sparse. Whiskers = 5–95 percentile, + = mean, and line = median. ***$p < 0.0001$, ns = not significant by unpaired, 2-tailed Mann-Whitney test. (**C**) Venn diagram of genes with a greater than 1.5-fold difference between dense and sparse ($p < 0.05$, two sample t-test, equal variance) common among NHAs and glioma TS lines. (**D**) Volcano plots of density-dependent genes in NHAs and glioma TS lines. White areas denote genes more than 1.5-fold different between sparse and dense with a significance of $p < 0.05$. (**E**) Bar chart of quantitative real time PCR for select cholesterol biosynthetic genes as shown in Figure 1G. Bars are the ratio of dense over sparse gene expression, normalized to GAPDH. Error bars are SEM from at least 3 biological replicates. (**F**) Bar chart of quantitative real time PCR for cholesterol regulation and transport genes. Error bars are SEM from at least 3 biological replicates. (**G**) Bar chart of quantitative real time PCR for select cholesterol biosynthetic genes in NHAs, WS1 normal lung fibroblasts, CCD-18Co normal colon myofibroblasts, HT29 and HCT116 colon cancer cells, or NCI-H522 and NCI-H3255 lung cancer cells. Bars are the ratio of dense over sparse gene expression, normalized to B2M. Error bars are SEM from at least 3 biological replicates. ns = not significant; *$p < 0.05$, ***$p < 0.001$, one-way ANOVA with Kruskal-Wallis and Dunn's multiple comparisons test for the average of all measured cholesterol synthesis genes for each cell line vs. NHA.

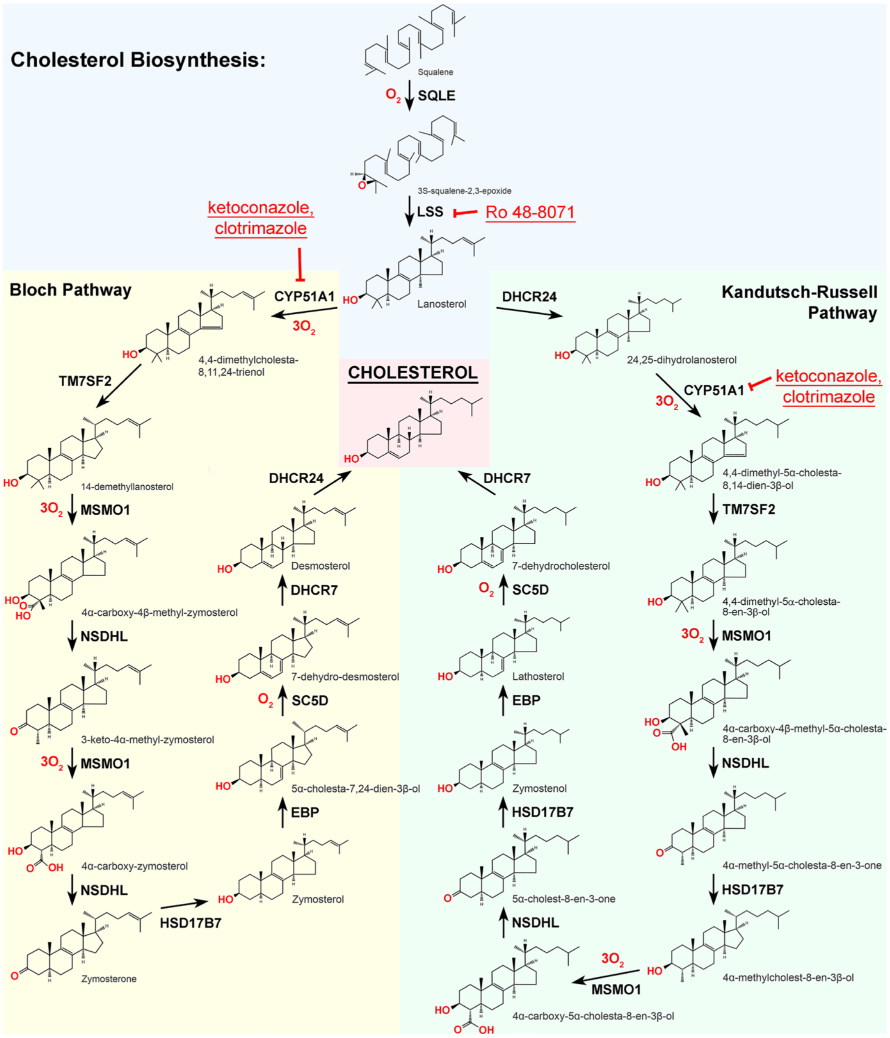

Supplementary Figure 2: The cholesterol biosynthesis pathway. Cholesterol synthesis can proceed from lanosterol down through one of two alternative pathways, the Bloch and Kandutsch-Russell pathways. These pathways use the same enzymes but differ in the order of the catalytic stages from lanosterol to cholesterol [6, 7]. Modified from humancyc.org, www.lipidmaps.org, and Reference 6. Ketoconazole and clotrimazole inhibit CYP51A1 and Ro 48-8071 inhibits LSS.

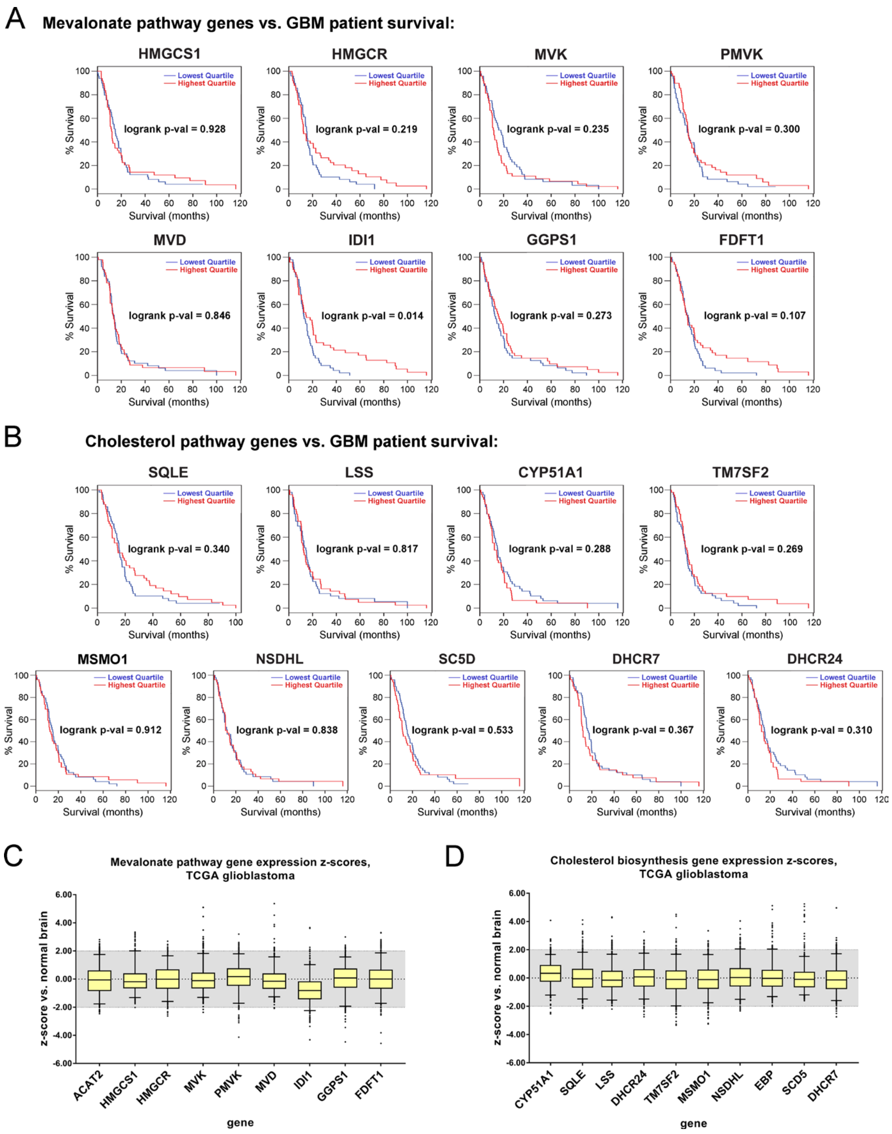

Supplementary Figure 3: Expression of individual genes in the mevalonate and cholesterol pathways is not significantly associated with poor prognosis for GBM patients. Kaplan-Meier curves for individual mevalonate (**A**) and cholesterol (**B**) biosynthesis pathway genes stratified by lowest vs. highest quartile gene expression. Data are from the TCGA. (**C**) Z-scores for mevalonate and biosynthesis genes for expression relative to normal brain. (**D**) Z-scores for cholesterol biosynthesis genes for expression relative to normal brain.

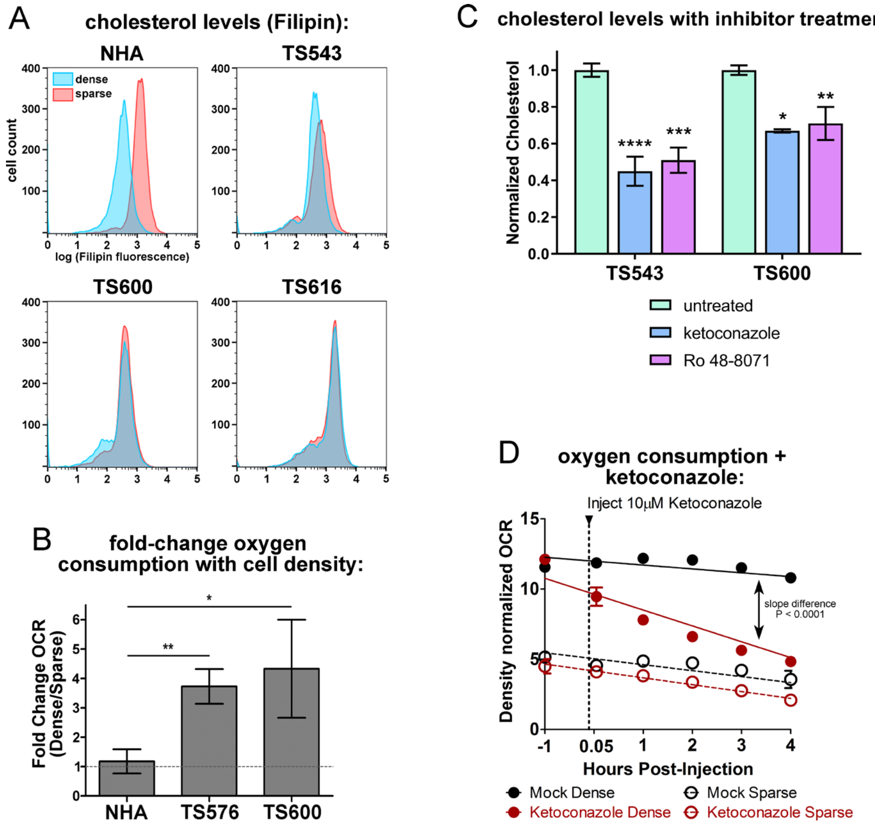

Supplementary Figure 4: Dense glioma cells use oxygen to maintain cholesterol synthesis. (**A**) Flow cytometry of sparse and dense NHA and glioma TS cells stained with the cholesterol stain, Filipin III. Curves represent 10,000 events. Dense cells were plated at 93,750 cells/cm^2 and sparse at 15,625 cells/cm^2 prior to harvesting for flow cytometry. Data shown are representative of at least 2 biological replicates. (**B**) Fold change in oxygen consumption rates (OCR) with density in NHA and glioma TS cells, expressed as dense relative to sparse. *p < 0.05, **p < 0.01, unpaired t-test, bars = SEM for 3 biological replicates. (**C**) Cholesterol quantitation in ketoconazole and Ro 48-8071-treated TS543 and TS600, expressed relative to untreated. Cells were treated with 5 μM ketoconazole or 1 μM Ro 48–8071 for 6 hours prior to cholesterol measurements. Bars = SEM for at least 2 biological replicates. *p < 0.05, **p < 0.01, ***p < 0.005, ****p < 0.0001, 2-way ANOVA without correction for multiple comparisons using a Fisher's LSD test. (**D**) OCR in mock- and ketoconazole-treated sparse and dense TS543 cells. OCR is normalized to DNA content to control for cell number. Bars = SD.

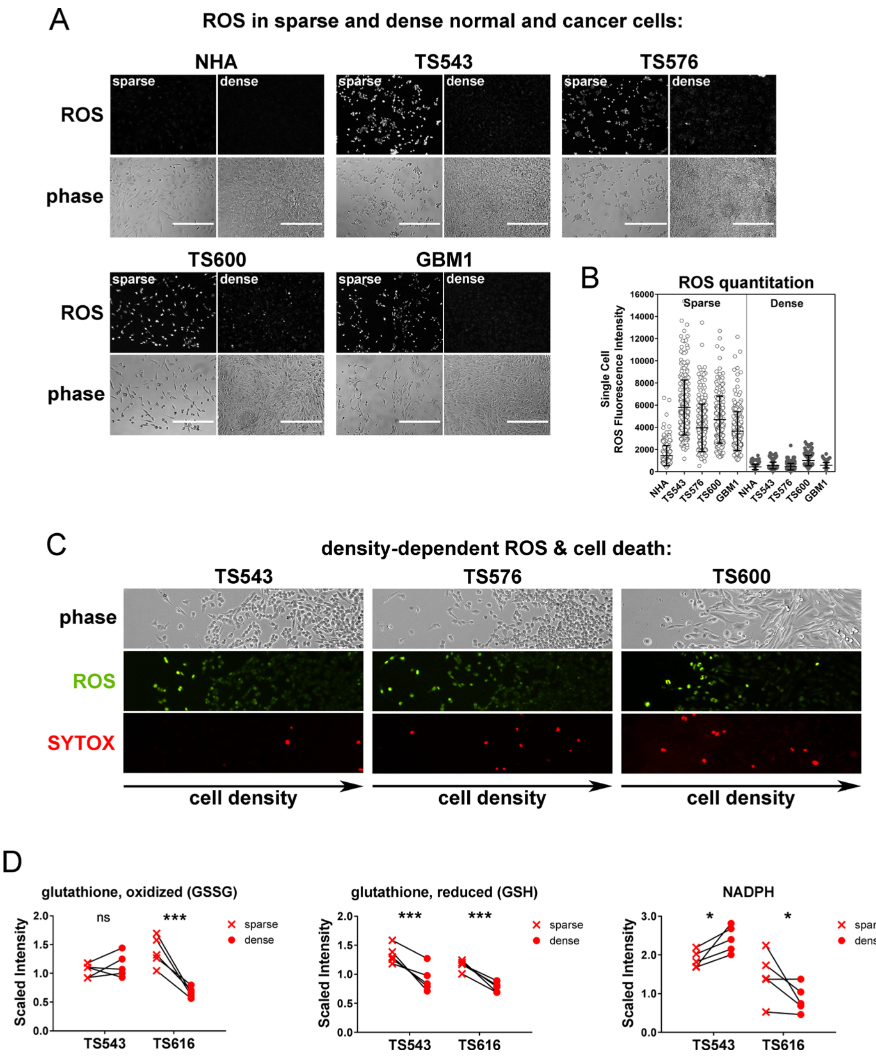

Supplementary Figure 5: Dense cells have decreased mitochondrial respiratory activity compared to sparse. (**A**) ROS staining in sparse and dense NHAs and TS glioma TS cells, Bar = 400 µm. Sparse = 15,625 cells/cm^2; Dense = 93,750 cells/cm^2. (**B**) Quantitation of ROS intensity of (**A**) Circle = ROS fluorescence intensity of a single cell. Bars = SD for at least 250 measured cells. Data shown are representative of at least 4 biological replicates. (**C**) ROS and cell death in glioma cells cultured on a density gradient. ROS was stained with CellROX Green and dead cells with SYTOX Orange. (**D**) Oxidized glutathione, reduced glutathione, and NADPH quantitation, with lines connecting paired sparse and dense biological replicates for each cell line. *$p \leq 0.1$, **$p \leq 0.001$, ***$p \leq 0.001$, ****$p \leq 0.0001$, ns = not significant by paired 2-Way ANOVA and Fisher's LSD test for 5 biological replicates.

A

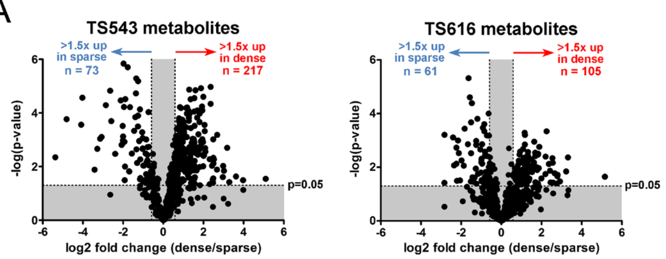

B

SUPER PATHWAY	TS543 1.5 fold up sparse	TS543 1.5 fold up dense	TS543 total metabolites	TS543 median fold change (log2(D/S))	TS543 P-value	TS616 1.5 fold up sparse	TS616 1.5 fold up dense	TS616 total metabolites	TS616 median fold change (log2(D/S))	TS616 P-value
Amino Acids	33	22	124	-0.04	0.1569	21	18	124	0.00	0.8429
Carbohydrates	0	24	39	1.07	**< 0.0001**	7	4	39	0.14	0.7115
Cofactors and Vitamins	4	5	27	0.02	0.5422	3	3	27	-0.03	0.8195
Energy	5	1	8	-1.04	0.0781	3	0	8	-0.49	**0.0078**
Lipids	21	95	196	0.55	**< 0.0001**	47	42	196	-0.07	0.3440
Nucleotides	9	23	54	0.37	**0.0076**	13	14	54	0.00	0.7500
Peptides	9	71	105	0.86	**< 0.0001**	3	84	105	1.08	**< 0.0001**
Xenobiotics	1	3	13	0.40	0.0771	3	4	13	0.09	0.1763

C

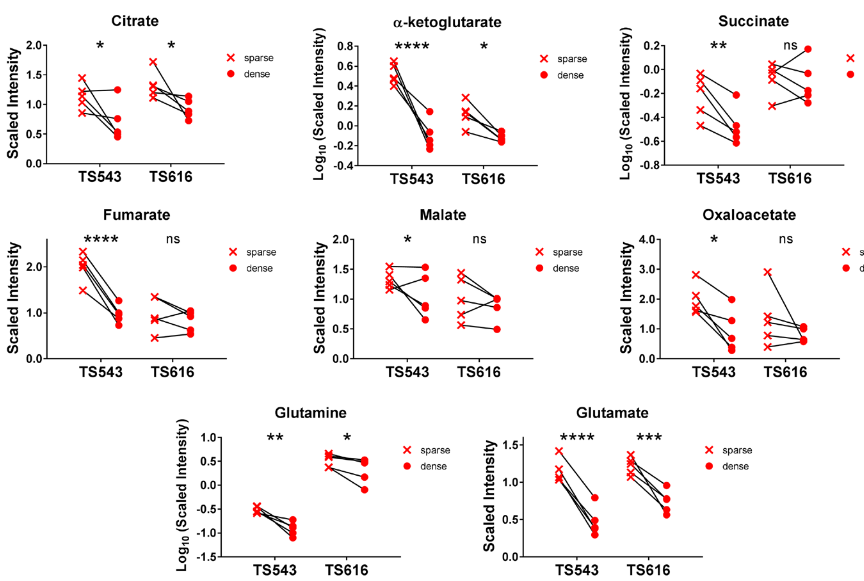

D

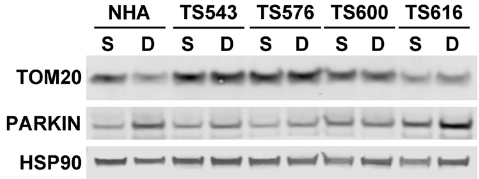

E

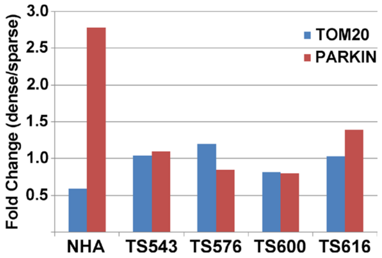

Supplementary Figure 6: Metabolic profiling of sparse and dense glioma TS543 and TS616 cells, and mitochondrial protein levels. (**A**) Volcano plots of density-dependent metabolites in TS543 and TS616 glioma cells. White areas denote metabolites more than 1.5 fold different between sparse and dense with a significance of $p < 0.05$. (**B**) Changes in metabolic super pathways in TS543 and TS616 glioma cells. Significant differences in the number of metabolites increased in sparse vs. dense was determined using a Wilcoxon Signed Rank Test. (**C**) Quantitation of TCA cycle metabolites depicted in Figure 4B, with lines connecting paired sparse and dense biological replicates for each cell line. *$p \leq 0.1$, **$p \leq 0.01$, ***$p \leq 0.001$, ****$p \leq 0.0001$, ns = not significant by paired 2-Way ANOVA and a Fisher's LSD test for 5 biological replicates for sparse vs dense. (**D**) Immunoblot of mitochondrial markers TOM20 and Parkin from whole cell lysates from sparse (S, 15,625 cells/cm^2) and dense (D, 93,750 cells/cm^2) NHAs and glioma TS cells. (**E**) Densitometry of immunoblot in C, normalized to loading control HSP90.

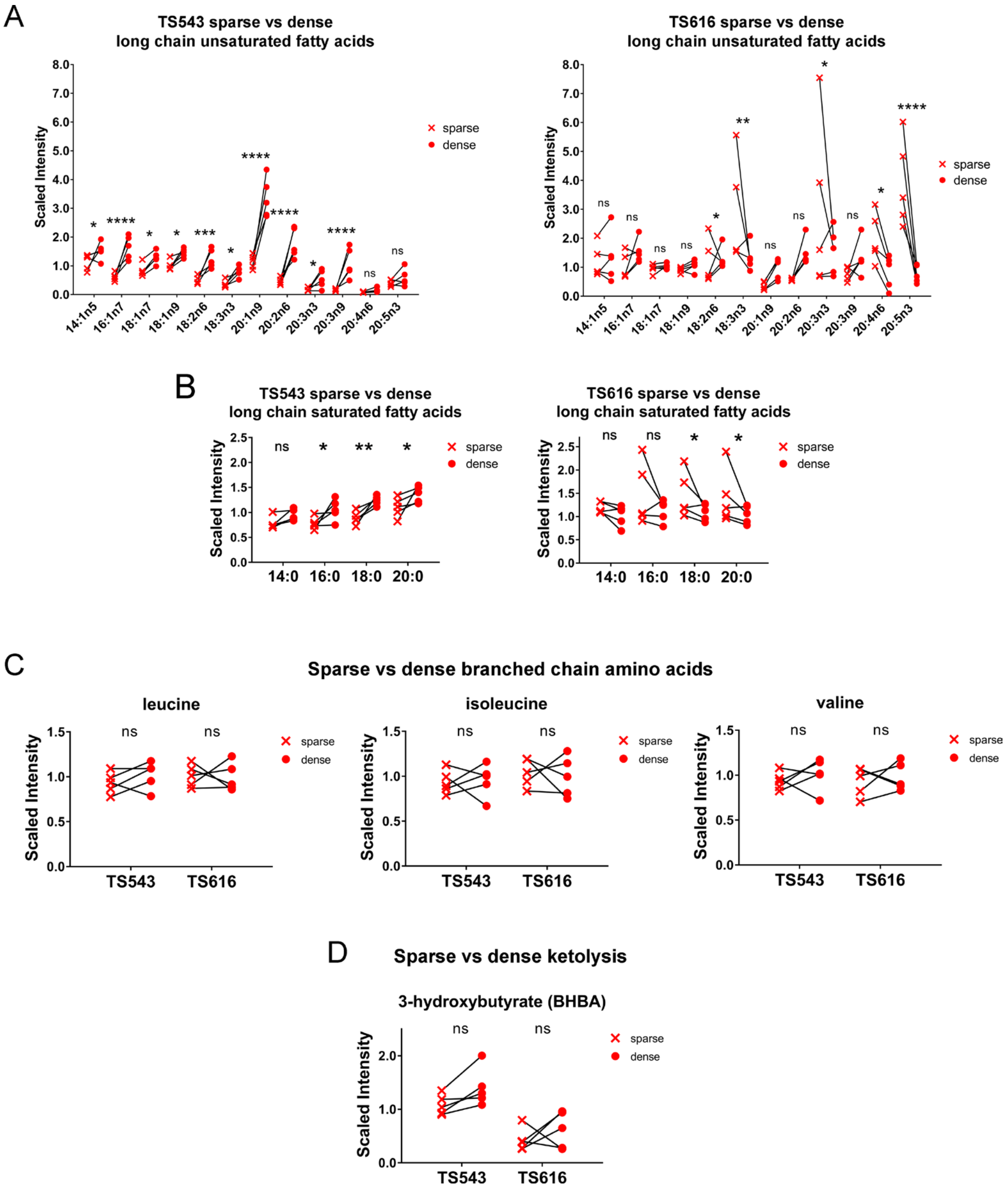

Supplementary Figure 7: Neither fatty acid oxidation, branched chain amino acid degradation, nor ketolysis are likely to be major sources of NADPH for cholesterol synthesis in dense cells. (**A**) Quantitation of long chain unsaturated fatty acids in sparse and dense TS543 (left) and TS616 (right) glioma TS cells. (**B**) Quantitation of long chain saturated fatty acids in sparse and dense TS543 (left) and TS616 (right) glioma TS cells. (**C**) Quantitation of branched chain amino acids leucine (left), isoleucine (center) and valine (right) in sparse and dense TS543 and TS616 glioma TS cells. (**D**) Quantitation of 3-hydroxybutyrate in sparse and dense TS543 and TS616 glioma TS cells. For all panels, *$p \leq 0.1$, **$p \leq 0.01$, ***$p \leq 0.001$, ****$p \leq 0.0001$, ns = not significant by paired 2-Way ANOVA and a Fisher's LSD test for 5 biological replicates for sparse vs dense.

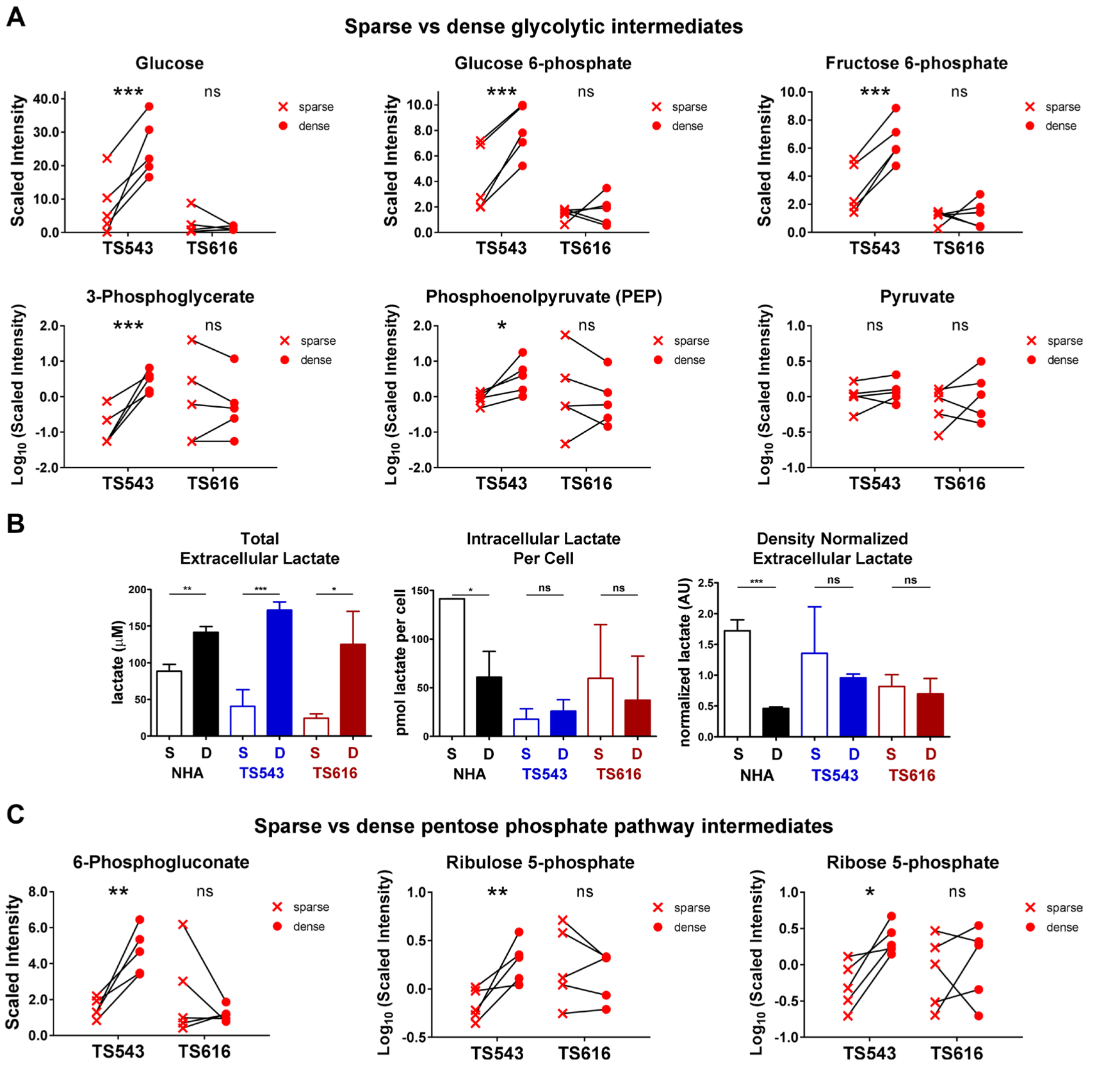

Supplementary Figure 8: High cell density leads to upregulation of aerobic glycolysis without an increase in lactate. (**A**) Quantitation of glycolysis metabolites depicted in Figure 4B, with lines connecting paired sparse and dense biological replicates for each cell line. *$p \leq 0.1$, **$p \leq 0.001$, ***$p \leq 0.001$, ****p ≤0.0001, ns = not significant by paired 2-Way ANOVA and a Fisher's LSD test for 5 biological replicates for sparse vs dense. Sparse = 15,625 cells/cm^2; Dense = 93,750 cells/cm^2. (**B**) Quantitation of total and density-normalized intracellular and extracellular lactate in sparse (S) and dense (D) NHAs and glioma TS cells. For cell density normalization, values were normalized to cell number. *$p \leq 0.05$, **$p \leq 0.002$, ***$p \leq 0.0003$, ns = not significant, unpaired *t*-test. Bars = SEM for 3 biological replicates. AU = arbitrary units. (**C**) Quantitation of pentose phosphate pathway intermediates in sparse and dense TS543 and TS616 glioma TS cells. *$p \leq 0.1$, **$p \leq 0.01$, ***$p \leq 0.001$, ****$p \leq 0.0001$, ns = not significant by paired 2-Way ANOVA and a Fisher's LSD test for 5 biological replicates for sparse vs dense.

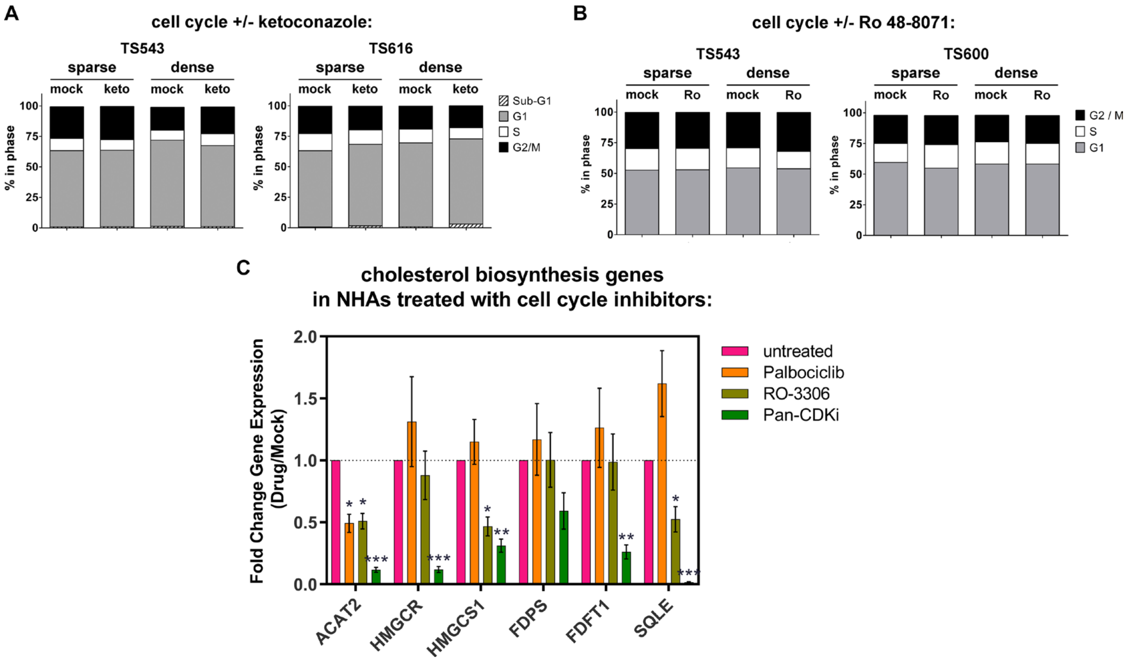

Supplementary Figure 9: Cell cycle analysis of sparse and dense glioma TS cells treated with cholesterol synthesis pathway inhibitors. Sparse cells were plated at 50,000 cells per mL and dense at 300,000. Cells were harvested 6 hours after treatment with DMSO (mock) or 5 μM ketoconazole (**A**) or 1 μM Ro 48-8071 (**B**). Data shown are representative of 2 biological replicates. (**C**) Bar chart of quantitative real time PCR for select cholesterol biosynthetic genes in low density NHAs treated with cell cycle inhibitors. Bars are the ratio of drug-treated over mock gene expression, normalized to GAPDH. Error bars are SEM from 3 (palbociclib) or 4 (RO-3306, pan-CDKi) biological replicates. *$p \leq 0.05$, **$p \leq 0.002$, ***$p \leq 0.0003$, 2-tailed t-test vs. a theoretical mean of 1. No asterisks = not significant.

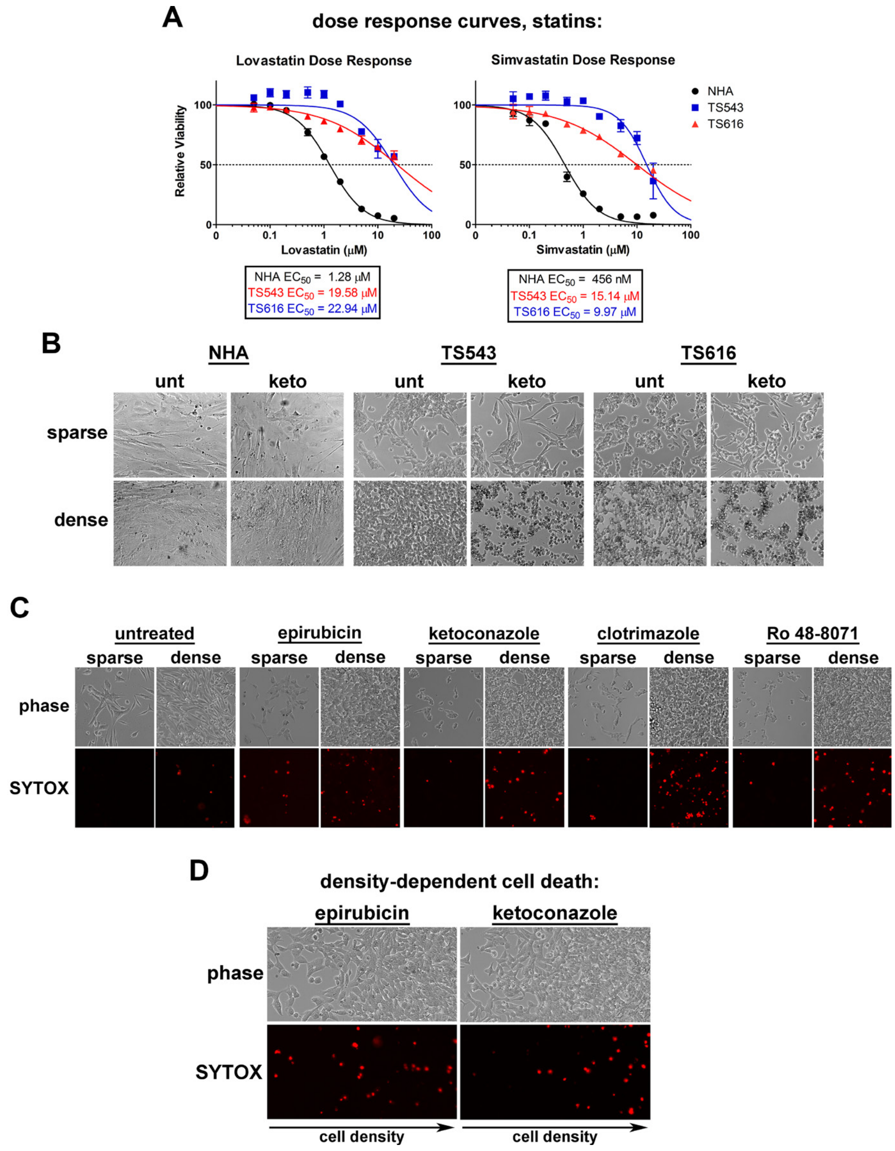

Supplementary Figure 10: Effects of cholesterol synthesis inhibitors on sparse and dense glioma cells. (**A**) Dose response curves for lovastatin (left) and simvastatin treated cells. Data shown are representative of at least 3 biological replicates. Bars = SD. (**B**) Representative phase contrast images from cells in Figure 6A. Cells were untreated or incubated with 5 μM ketoconazole for 72 hrs. (**C**) Cell death in TS600 glioma cells. Dead cells were visualized with SYTOX Orange 24 hours after treatment with DMSO (mock), 1 μM epirubicin, 10 μM clotrimazole, 5 μM ketoconazole, or 1 μM Ro 48-8071. Sparse = 15,625 cells/cm^2; dense = 93,750 cells/cm^2. (**D**) Cell death in TS600 glioma cells cultured on a density gradient. Dead cells were visualized with SYTOX Orange 24 hours after treatment with DMSO (mock) (see Supplementary Figure 5C), 1 μM epirubicin, or 5 μM ketoconazole.

Supplementary Table 1: List of genes in cluster 8 from Self-Organizing Map (Figure 5A).
See Supplementary _Table_1
