# Supplementary material for: Disabled cell density sensing leads to dysregulated cholesterol synthesis in glioblastoma
Source: Oncotarget. 2017 Jan 19;8(9):14860–75. doi: 10.18632/oncotarget.14740 (PMC5362450; doi:10.18632/oncotarget.14740)
Supplement: Supplementary file 2 [file oncotarget-08-14860-s002.docx]

Table S1

| Cluster ID | Row | Column | gene symbol | log2 (fold change dense/sparse) NHA | log2 (fold change dense/sparse) TS543 | log2 (fold change dense/sparse) TS576 | log2 (fold change dense/sparse) TS616 |
| --- | --- | --- | --- | --- | --- | --- | --- |
| 8 | 1 | 8 | ACPL2 | -0.68 | 0.10 | -0.05 | 0.36 |
| 8 | 1 | 8 | ACSL3 | -0.66 | 0.02 | 0.13 | 0.08 |
| 8 | 1 | 8 | ACTA2 | -1.06 | 0.11 | 0.13 | 0.22 |
| 8 | 1 | 8 | ACTBL2 | -0.92 | -0.03 | 0.08 | -0.16 |
| 8 | 1 | 8 | ADAM19 | -0.71 | 0.10 | 0.18 | -0.03 |
| 8 | 1 | 8 | ADAMTS9 | -0.66 | 0.25 | 0.45 | 0.46 |
| 8 | 1 | 8 | ADI1 | -0.65 | 0.12 | -0.28 | -0.15 |
| 8 | 1 | 8 | ADRB2 | -0.84 | 0.01 | 0.14 | -0.09 |
| 8 | 1 | 8 | AFP | -0.75 | -0.02 | -0.16 | -0.08 |
| 8 | 1 | 8 | AGPAT9 | -1.61 | 0.06 | 0.07 | 0.16 |
| 8 | 1 | 8 | AIG1 | -0.59 | 0.27 | 0.03 | 0.23 |
| 8 | 1 | 8 | AJUBA | -0.62 | 0.18 | 0.06 | -0.05 |
| 8 | 1 | 8 | ALCAM | -0.67 | -0.27 | -0.21 | 0.17 |
| 8 | 1 | 8 | ALPK2 | -1.05 | 0.47 | 0.47 | 0.04 |
| 8 | 1 | 8 | AMIGO2 | -0.75 | 0.09 | 0.18 | 0.22 |
| 8 | 1 | 8 | AMOTL2 | -0.65 | -0.25 | 0.33 | -0.25 |
| 8 | 1 | 8 | ANK1 | -0.80 | -0.08 | -0.26 | -0.29 |
| 8 | 1 | 8 | ANKRD1 | -1.17 | -0.01 | 0.12 | -0.05 |
| 8 | 1 | 8 | ANKS1B | -1.00 | 0.07 | -0.10 | 0.27 |
| 8 | 1 | 8 | ANLN | -1.27 | -0.34 | -0.30 | -0.09 |
| 8 | 1 | 8 | ANO4 | -0.92 | 0.08 | 0.00 | -0.05 |
| 8 | 1 | 8 | ANXA3 | -1.12 | -0.09 | -0.09 | -0.24 |
| 8 | 1 | 8 | AP1M2 | -0.65 | 0.04 | 0.05 | 0.03 |
| 8 | 1 | 8 | APOBEC3B | -0.81 | 0.14 | -0.05 | 0.18 |
| 8 | 1 | 8 | ARHGAP11A | -0.74 | -0.28 | -0.42 | 0.35 |
| 8 | 1 | 8 | ARHGAP18 | -0.63 | 0.32 | -0.38 | -0.21 |
| 8 | 1 | 8 | ARHGAP22 | -0.81 | -0.18 | -0.03 | -0.30 |
| 8 | 1 | 8 | ARHGAP29 | -0.55 | 0.10 | 0.09 | 0.09 |
| 8 | 1 | 8 | ARSJ | -0.62 | -0.05 | 0.05 | 0.35 |
| 8 | 1 | 8 | ASB9 | -0.55 | -0.10 | 0.12 | -0.15 |
| 8 | 1 | 8 | ASPM | -0.68 | -0.02 | 0.06 | 0.07 |
| 8 | 1 | 8 | ATF7IP | -0.58 | -0.21 | -0.05 | -0.20 |
| 8 | 1 | 8 | AURKA | -0.82 | -0.10 | -0.13 | -0.02 |
| 8 | 1 | 8 | AURKB | -0.73 | -0.12 | -0.25 | -0.05 |
| 8 | 1 | 8 | BAMBI | -0.84 | 0.26 | 0.01 | 0.23 |
| 8 | 1 | 8 | BDNF | -1.35 | -0.12 | 0.17 | -0.24 |
| 8 | 1 | 8 | BORA | -0.55 | -0.04 | -0.18 | 0.08 |
| 8 | 1 | 8 | BPGM | -0.57 | 0.07 | 0.01 | 0.19 |
| 8 | 1 | 8 | BUB1 | -0.69 | -0.03 | -0.01 | 0.07 |
| 8 | 1 | 8 | C10orf54 | -0.61 | 0.09 | -0.09 | 0.08 |
| 8 | 1 | 8 | C15orf52 | -0.75 | 0.50 | 0.19 | 0.10 |
| 8 | 1 | 8 | CCDC107 | -0.51 | 0.07 | -0.14 | -0.09 |
| 8 | 1 | 8 | CCDC81 | -0.93 | 0.13 | 0.15 | 0.05 |
| 8 | 1 | 8 | CCDC85C | -0.62 | 0.07 | -0.17 | -0.09 |
| 8 | 1 | 8 | CCNA2 | -0.78 | -0.22 | -0.28 | 0.09 |
| 8 | 1 | 8 | CCNB1 | -0.70 | -0.18 | -0.35 | 0.02 |
| 8 | 1 | 8 | CCNB2 | -0.58 | 0.01 | -0.06 | 0.05 |
| 8 | 1 | 8 | CCND1 | -1.05 | 0.13 | 0.20 | -0.31 |
| 8 | 1 | 8 | CD274 | -0.60 | -0.08 | 0.21 | -0.07 |
| 8 | 1 | 8 | CDC25C | -0.65 | 0.15 | -0.01 | 0.02 |
| 8 | 1 | 8 | CDCA3 | -0.83 | 0.04 | -0.06 | 0.04 |
| 8 | 1 | 8 | CDCA8 | -0.77 | -0.20 | -0.19 | 0.15 |
| 8 | 1 | 8 | CDH13 | -0.57 | 0.48 | -0.29 | 0.25 |
| 8 | 1 | 8 | CDKN2B | -1.19 | 1.01 | 0.11 | -0.08 |
| 8 | 1 | 8 | CENPA | -0.61 | 0.21 | 0.04 | 0.13 |
| 8 | 1 | 8 | CENPF | -0.64 | -0.25 | -0.26 | 0.07 |
| 8 | 1 | 8 | CENPH | -0.55 | 0.06 | -0.03 | 0.07 |
| 8 | 1 | 8 | CEP55 | -0.97 | -0.16 | -0.26 | 0.10 |
| 8 | 1 | 8 | CGNL1 | -0.70 | -0.08 | 0.11 | 0.30 |
| 8 | 1 | 8 | CHST7 | -0.62 | -0.08 | -0.02 | -0.41 |
| 8 | 1 | 8 | CKAP2L | -0.63 | 0.08 | -0.18 | 0.12 |
| 8 | 1 | 8 | CKMT2 | -0.58 | 0.13 | -0.06 | -0.06 |
| 8 | 1 | 8 | CKS2 | -0.49 | 0.15 | 0.03 | 0.11 |
| 8 | 1 | 8 | CLDN22 | -0.85 | -0.04 | -0.10 | 0.12 |
| 8 | 1 | 8 | CNIH3 | -0.73 | 0.41 | 0.29 | 0.49 |
| 8 | 1 | 8 | CNN1 | -0.74 | 0.11 | 0.24 | -0.12 |
| 8 | 1 | 8 | COL4A5 | -1.02 | 0.32 | 0.48 | 0.23 |
| 8 | 1 | 8 | COL4A6 | -1.00 | 0.65 | -0.23 | -0.27 |
| 8 | 1 | 8 | COTL1 | -0.70 | -0.09 | -0.35 | -0.12 |
| 8 | 1 | 8 | CRHBP | -1.57 | -0.03 | 0.18 | 0.06 |
| 8 | 1 | 8 | CRIM1 | -1.06 | -0.03 | 0.22 | -0.09 |
| 8 | 1 | 8 | CTNNAL1 | -0.65 | -0.10 | -0.27 | 0.11 |
| 8 | 1 | 8 | CYS1 | -0.64 | 0.15 | 0.15 | 0.39 |
| 8 | 1 | 8 | CYTL1 | -0.82 | 0.28 | -0.15 | -0.54 |
| 8 | 1 | 8 | DAB1 | -0.73 | -0.23 | 0.44 | -0.25 |
| 8 | 1 | 8 | DCBLD2 | -1.11 | -0.11 | -0.15 | -0.04 |
| 8 | 1 | 8 | DEPDC1 | -1.00 | -0.40 | -0.22 | 0.22 |
| 8 | 1 | 8 | DEPDC1B | -0.73 | -0.16 | -0.39 | 0.13 |
| 8 | 1 | 8 | DHCR7 | -0.91 | -0.14 | -0.32 | -0.34 |
| 8 | 1 | 8 | DIAPH3 | -0.87 | -0.11 | -0.02 | 0.10 |
| 8 | 1 | 8 | DKK1 | -1.83 | -0.01 | 0.14 | 0.01 |
| 8 | 1 | 8 | DLG1 | -0.60 | -0.13 | -0.25 | 0.16 |
| 8 | 1 | 8 | DLGAP5 | -0.74 | -0.02 | -0.06 | 0.20 |
| 8 | 1 | 8 | DOCK2 | -0.86 | -0.04 | -0.17 | -0.10 |
| 8 | 1 | 8 | DOCK5 | -0.64 | 0.10 | 0.16 | -0.19 |
| 8 | 1 | 8 | DRAXIN | -0.61 | 0.30 | 0.39 | 0.31 |
| 8 | 1 | 8 | DUSP4 | -0.57 | 0.36 | -0.23 | 0.00 |
| 8 | 1 | 8 | EBP | -0.55 | -0.07 | -0.14 | -0.23 |
| 8 | 1 | 8 | EDN1 | -1.41 | 0.19 | 0.11 | 0.02 |
| 8 | 1 | 8 | EGF | -1.45 | -0.09 | 0.13 | 0.13 |
| 8 | 1 | 8 | EML6 | -0.78 | -0.12 | 0.07 | 0.03 |
| 8 | 1 | 8 | EN1 | -0.73 | 0.16 | 0.11 | -0.02 |
| 8 | 1 | 8 | ERRFI1 | -0.62 | 0.14 | 0.02 | -0.07 |
| 8 | 1 | 8 | FADS2 | -0.74 | 0.02 | -0.39 | -0.11 |
| 8 | 1 | 8 | FAHD2A | -0.69 | 0.15 | -0.04 | 0.07 |
| 8 | 1 | 8 | FAM64A | -0.78 | -0.16 | -0.14 | 0.02 |
| 8 | 1 | 8 | FAM83D | -0.99 | -0.24 | -0.30 | -0.06 |
| 8 | 1 | 8 | FAM84B | -0.58 | -0.03 | 0.12 | -0.03 |
| 8 | 1 | 8 | FDFT1 | -0.70 | 0.20 | -0.03 | -0.17 |
| 8 | 1 | 8 | FDPS | -0.72 | 0.10 | 0.04 | -0.09 |
| 8 | 1 | 8 | FGF5 | -0.73 | 0.08 | 0.22 | 0.18 |
| 8 | 1 | 8 | FLG | -0.72 | 0.06 | 0.19 | -0.07 |
| 8 | 1 | 8 | FLJ42393 | -0.63 | -0.10 | 0.05 | -0.38 |
| 8 | 1 | 8 | FOSL1 | -1.26 | -0.23 | 0.27 | -0.32 |
| 8 | 1 | 8 | FOXD1 | -1.18 | 0.25 | 0.14 | -0.07 |
| 8 | 1 | 8 | FOXF1 | -1.10 | 0.25 | -0.08 | -0.02 |
| 8 | 1 | 8 | FOXN3 | -0.70 | 0.18 | 0.19 | 0.05 |
| 8 | 1 | 8 | FREM2 | -0.60 | 0.27 | -0.08 | 0.36 |
| 8 | 1 | 8 | FSTL1 | -0.66 | -0.05 | 0.15 | 0.25 |
| 8 | 1 | 8 | GABRG3 | -0.64 | -0.07 | -0.08 | -0.14 |
| 8 | 1 | 8 | GAS6 | -0.59 | 0.14 | -0.11 | 0.04 |
| 8 | 1 | 8 | GATA6 | -0.85 | -0.04 | -0.03 | -0.10 |
| 8 | 1 | 8 | GBP5 | -0.59 | 0.14 | 0.11 | 0.09 |
| 8 | 1 | 8 | GDPD5 | -0.71 | 0.04 | -0.06 | 0.08 |
| 8 | 1 | 8 | GLS | -0.58 | 0.10 | -0.20 | -0.28 |
| 8 | 1 | 8 | GOPC | -0.75 | 0.13 | 0.13 | 0.09 |
| 8 | 1 | 8 | GPR18 | -1.13 | 0.40 | 0.39 | 0.11 |
| 8 | 1 | 8 | GREM1 | -1.71 | 0.06 | -0.12 | -0.11 |
| 8 | 1 | 8 | GRIA1 | -0.73 | -0.05 | 0.09 | 0.18 |
| 8 | 1 | 8 | GRPR | -1.11 | 0.01 | 0.07 | 0.05 |
| 8 | 1 | 8 | HDAC9 | -0.59 | 0.18 | 0.22 | 0.50 |
| 8 | 1 | 8 | HEG1 | -1.05 | 0.12 | 0.15 | -0.12 |
| 8 | 1 | 8 | HJURP | -0.70 | -0.19 | -0.23 | -0.15 |
| 8 | 1 | 8 | HLF | -0.69 | -0.31 | 0.10 | 0.12 |
| 8 | 1 | 8 | HMGA1 | -1.05 | 0.16 | -0.31 | 0.18 |
| 8 | 1 | 8 | HMGCS1 | -0.71 | -0.08 | -0.21 | 0.01 |
| 8 | 1 | 8 | HMMR | -0.69 | -0.18 | -0.19 | 0.08 |
| 8 | 1 | 8 | HOXB2 | -0.69 | 0.00 | 0.07 | -0.13 |
| 8 | 1 | 8 | HOXB3 | -0.77 | -0.07 | 0.16 | -0.22 |
| 8 | 1 | 8 | HOXB5 | -1.18 | 0.22 | 0.13 | 0.17 |
| 8 | 1 | 8 | IDI1 | -0.74 | 0.05 | 0.10 | -0.11 |
| 8 | 1 | 8 | IER3 | -0.76 | -0.11 | -0.04 | -0.06 |
| 8 | 1 | 8 | IFFO2 | -0.77 | 0.08 | -0.06 | -0.27 |
| 8 | 1 | 8 | IGF2 | -0.94 | 0.44 | 0.15 | 0.09 |
| 8 | 1 | 8 | IGFBP4 | -1.19 | 0.12 | -0.36 | 0.19 |
| 8 | 1 | 8 | IGFBP6 | -0.84 | 0.10 | -0.19 | 0.04 |
| 8 | 1 | 8 | IL18 | -1.87 | -0.15 | -0.23 | -0.18 |
| 8 | 1 | 8 | IL6 | -1.24 | -0.10 | 0.26 | 0.05 |
| 8 | 1 | 8 | INA | -0.67 | 0.17 | 0.21 | 0.00 |
| 8 | 1 | 8 | INSIG1 | -1.05 | 0.16 | 0.19 | 0.03 |
| 8 | 1 | 8 | IRX1 | -0.60 | 0.04 | -0.08 | 0.05 |
| 8 | 1 | 8 | IRX3 | -0.71 | 0.10 | 0.21 | 0.03 |
| 8 | 1 | 8 | ITGA3 | -0.93 | 0.36 | 0.29 | 0.25 |
| 8 | 1 | 8 | IVNS1ABP | -0.64 | 0.07 | -0.32 | -0.10 |
| 8 | 1 | 8 | KATNBL1 | -0.74 | -0.10 | -0.16 | -0.34 |
| 8 | 1 | 8 | KCNH1 | -0.61 | 0.09 | 0.13 | 0.07 |
| 8 | 1 | 8 | KCNH2 | -0.84 | 0.22 | -0.04 | -0.22 |
| 8 | 1 | 8 | KCNJ2 | -1.51 | -0.05 | 0.07 | -0.18 |
| 8 | 1 | 8 | KCNJ6 | -0.77 | -0.15 | 0.19 | 0.09 |
| 8 | 1 | 8 | KCNK1 | -0.75 | 0.28 | -0.28 | 0.04 |
| 8 | 1 | 8 | KCNK2 | -1.12 | -0.18 | -0.07 | 0.14 |
| 8 | 1 | 8 | KDR | -1.05 | 0.01 | 0.13 | 0.25 |
| 8 | 1 | 8 | KIAA1804 | -1.05 | -0.16 | 0.17 | -0.23 |
| 8 | 1 | 8 | KIF18A | -0.65 | -0.03 | -0.23 | 0.12 |
| 8 | 1 | 8 | KIF20A | -0.70 | 0.00 | -0.17 | 0.10 |
| 8 | 1 | 8 | KIF20B | -0.61 | -0.17 | -0.16 | -0.06 |
| 8 | 1 | 8 | KIF23 | -0.73 | -0.14 | -0.13 | 0.05 |
| 8 | 1 | 8 | KIF2C | -0.73 | -0.14 | -0.22 | -0.07 |
| 8 | 1 | 8 | KIFC1 | -0.61 | -0.06 | -0.36 | 0.02 |
| 8 | 1 | 8 | KIRREL3 | -0.68 | 0.16 | 0.12 | -0.14 |
| 8 | 1 | 8 | KLF7 | -0.64 | -0.26 | 0.09 | -0.27 |
| 8 | 1 | 8 | KNSTRN | -0.73 | -0.08 | -0.20 | -0.03 |
| 8 | 1 | 8 | KRT18 | -0.68 | -0.13 | -0.05 | -0.11 |
| 8 | 1 | 8 | KRT80 | -1.16 | 0.07 | 0.12 | -0.05 |
| 8 | 1 | 8 | LAYN | -1.05 | -0.18 | 0.23 | 0.28 |
| 8 | 1 | 8 | LBH | -0.78 | 0.33 | 0.13 | 0.09 |
| 8 | 1 | 8 | LDB3 | -0.84 | 0.08 | 0.14 | 0.08 |
| 8 | 1 | 8 | LDLR | -0.99 | -0.13 | 0.14 | -0.14 |
| 8 | 1 | 8 | LINC00472 | -0.77 | -0.03 | -0.07 | -0.20 |
| 8 | 1 | 8 | LIX1 | -0.94 | 0.08 | 0.20 | -0.03 |
| 8 | 1 | 8 | LMO7 | -0.92 | 0.18 | -0.20 | 0.26 |
| 8 | 1 | 8 | MATN2 | -1.05 | -0.30 | -0.12 | 0.44 |
| 8 | 1 | 8 | MROH1 | -0.65 | 0.14 | -0.23 | 0.12 |
| 8 | 1 | 8 | LPCAT2 | -1.00 | 0.19 | 0.25 | -0.13 |
| 8 | 1 | 8 | LPIN1 | -0.70 | 0.16 | 0.34 | -0.18 |
| 8 | 1 | 8 | LPP | -0.62 | -0.23 | 0.14 | -0.06 |
| 8 | 1 | 8 | LRP8 | -0.82 | -0.31 | -0.16 | 0.15 |
| 8 | 1 | 8 | LRRC3 | -0.65 | 0.12 | 0.25 | 0.21 |
| 8 | 1 | 8 | LYPD6 | -1.17 | -0.33 | -0.07 | 0.15 |
| 8 | 1 | 8 | LYPD6B | -2.13 | 0.01 | 0.05 | 0.01 |
| 8 | 1 | 8 | MALT1 | -0.64 | 0.40 | 0.18 | 0.16 |
| 8 | 1 | 8 | MARCH4 | -1.46 | -0.06 | 0.02 | -0.19 |
| 8 | 1 | 8 | MBP | -0.78 | 0.23 | 0.27 | -0.04 |
| 8 | 1 | 8 | MCAM | -0.62 | -0.19 | 0.16 | -0.20 |
| 8 | 1 | 8 | MELK | -0.76 | -0.25 | -0.18 | -0.07 |
| 8 | 1 | 8 | MFAP5 | -0.96 | 0.13 | 0.21 | 0.06 |
| 8 | 1 | 8 | MICAL3 | -0.75 | -0.40 | 0.26 | -0.29 |
| 8 | 1 | 8 | MICB | -0.60 | 0.14 | 0.12 | -0.07 |
| 8 | 1 | 8 | MMP10 | -0.66 | 0.05 | 0.01 | -0.09 |
| 8 | 1 | 8 | MSMO1 | -0.65 | 0.14 | -0.28 | -0.22 |
| 8 | 1 | 8 | MST4 | -0.88 | -0.06 | 0.11 | -0.06 |
| 8 | 1 | 8 | MTFR2 | -0.79 | -0.30 | -0.26 | -0.06 |
| 8 | 1 | 8 | MYC | -1.13 | 0.06 | -0.06 | -0.02 |
| 8 | 1 | 8 | MYO10 | -0.87 | 0.17 | 0.17 | -0.03 |
| 8 | 1 | 8 | NANOS1 | -0.97 | 0.40 | 0.26 | 0.25 |
| 8 | 1 | 8 | NCEH1 | -1.13 | -0.18 | -0.14 | -0.06 |
| 8 | 1 | 8 | NDC80 | -0.53 | 0.00 | -0.08 | 0.02 |
| 8 | 1 | 8 | NDE1 | -0.70 | 0.35 | -0.25 | -0.03 |
| 8 | 1 | 8 | NDUFS2 | -0.62 | 0.13 | -0.10 | -0.36 |
| 8 | 1 | 8 | NEBL | -0.77 | -0.07 | 0.19 | 0.06 |
| 8 | 1 | 8 | NEFL | -1.02 | -0.05 | -0.12 | 0.14 |
| 8 | 1 | 8 | NEFM | -1.45 | 0.08 | 0.13 | -0.13 |
| 8 | 1 | 8 | NKX6-2 | -0.91 | 0.12 | 0.14 | -0.05 |
| 8 | 1 | 8 | NMNAT2 | -0.67 | 0.29 | 0.36 | 0.25 |
| 8 | 1 | 8 | NREP | -0.65 | 0.28 | 0.29 | 0.12 |
| 8 | 1 | 8 | NRK | -0.61 | 0.03 | -0.18 | -0.13 |
| 8 | 1 | 8 | NT5E | -0.68 | -0.27 | 0.19 | -0.22 |
| 8 | 1 | 8 | NTF3 | -0.99 | 0.04 | 0.22 | 0.12 |
| 8 | 1 | 8 | NTN4 | -0.62 | -0.15 | 0.11 | 0.07 |
| 8 | 1 | 8 | NUF2 | -0.71 | 0.24 | 0.08 | 0.11 |
| 8 | 1 | 8 | NUSAP1 | -0.54 | 0.07 | -0.16 | 0.20 |
| 8 | 1 | 8 | OCA2 | -1.01 | 0.28 | 0.18 | 0.02 |
| 8 | 1 | 8 | OLFML3 | -1.12 | -0.15 | 0.14 | -0.13 |
| 8 | 1 | 8 | OXTR | -1.19 | -0.31 | -0.19 | -0.36 |
| 8 | 1 | 8 | PALM2 | -1.25 | -0.10 | 0.31 | -0.35 |
| 8 | 1 | 8 | PBK | -0.76 | -0.08 | -0.21 | 0.15 |
| 8 | 1 | 8 | PBX3 | -0.62 | 0.03 | -0.26 | -0.12 |
| 8 | 1 | 8 | PCSK9 | -1.07 | -0.14 | -0.14 | -0.13 |
| 8 | 1 | 8 | PDCD1LG2 | -0.91 | -0.19 | -0.07 | -0.10 |
| 8 | 1 | 8 | PDE1C | -0.99 | 0.19 | 0.15 | 0.45 |
| 8 | 1 | 8 | PDGFB | -0.68 | 0.08 | 0.19 | -0.11 |
| 8 | 1 | 8 | PDLIM1 | -0.74 | 0.34 | 0.33 | 0.02 |
| 8 | 1 | 8 | PDLIM7 | -0.78 | 0.17 | 0.16 | 0.15 |
| 8 | 1 | 8 | PHLDA2 | -1.19 | 0.06 | 0.08 | 0.21 |
| 8 | 1 | 8 | PICALM | -0.64 | -0.17 | -0.19 | -0.10 |
| 8 | 1 | 8 | PITPNM3 | -0.83 | -0.06 | 0.19 | -0.07 |
| 8 | 1 | 8 | PKIB | -0.80 | 0.37 | -0.19 | 0.33 |
| 8 | 1 | 8 | PLA2G3 | -0.84 | 0.30 | 0.29 | -0.31 |
| 8 | 1 | 8 | PLAUR | -0.67 | 0.11 | -0.18 | 0.22 |
| 8 | 1 | 8 | PLEKHG4 | -0.62 | 0.00 | -0.18 | 0.13 |
| 8 | 1 | 8 | PLK2 | -0.89 | 0.16 | -0.21 | 0.22 |
| 8 | 1 | 8 | PNPLA3 | -1.12 | 0.02 | 0.29 | -0.37 |
| 8 | 1 | 8 | POLR1E | -0.66 | 0.09 | 0.04 | -0.07 |
| 8 | 1 | 8 | PPM1K | -0.70 | -0.15 | -0.25 | -0.06 |
| 8 | 1 | 8 | PRELID2 | -0.58 | 0.22 | 0.23 | 0.13 |
| 8 | 1 | 8 | PRKAG2 | -0.93 | 0.33 | 0.12 | -0.28 |
| 8 | 1 | 8 | PROCR | -0.62 | 0.06 | 0.20 | -0.01 |
| 8 | 1 | 8 | PRPS1 | -0.49 | 0.10 | -0.12 | -0.10 |
| 8 | 1 | 8 | PRR11 | -0.75 | -0.13 | -0.11 | 0.10 |
| 8 | 1 | 8 | PRR5L | -1.08 | 0.24 | 0.24 | 0.48 |
| 8 | 1 | 8 | PSG5 | -0.59 | 0.02 | 0.11 | 0.01 |
| 8 | 1 | 8 | PTGER4 | -0.74 | 0.09 | 0.24 | -0.25 |
| 8 | 1 | 8 | PTPLA | -0.69 | 0.34 | 0.08 | -0.17 |
| 8 | 1 | 8 | PVRL3 | -0.72 | 0.15 | 0.04 | 0.34 |
| 8 | 1 | 8 | RAB32 | -0.51 | 0.34 | -0.12 | 0.09 |
| 8 | 1 | 8 | RACGAP1 | -0.66 | -0.10 | -0.33 | 0.00 |
| 8 | 1 | 8 | RAPH1 | -0.82 | 0.12 | -0.36 | -0.20 |
| 8 | 1 | 8 | RARB | -0.68 | -0.08 | 0.25 | 0.15 |
| 8 | 1 | 8 | RGS20 | -1.24 | 0.00 | 0.09 | -0.05 |
| 8 | 1 | 8 | RGS5 | -0.79 | 0.31 | 0.14 | 0.08 |
| 8 | 1 | 8 | RMDN2 | -0.62 | -0.22 | -0.18 | -0.10 |
| 8 | 1 | 8 | RNF144B | -0.91 | -0.51 | 0.30 | -0.19 |
| 8 | 1 | 8 | RPL27A | -0.82 | -0.20 | -0.06 | -0.39 |
| 8 | 1 | 8 | RPRM | -0.90 | 0.06 | 0.18 | 0.22 |
| 8 | 1 | 8 | RRAS2 | -0.75 | -0.30 | -0.21 | 0.13 |
| 8 | 1 | 8 | RSPO3 | -0.89 | 0.06 | 0.24 | 0.12 |
| 8 | 1 | 8 | RYR2 | -1.07 | 0.16 | 0.24 | -0.05 |
| 8 | 1 | 8 | SCD | -1.09 | 0.13 | 0.21 | -0.21 |
| 8 | 1 | 8 | SCNN1A | -0.84 | 0.07 | 0.20 | -0.04 |
| 8 | 1 | 8 | SDPR | -0.69 | 0.04 | 0.29 | -0.02 |
| 8 | 1 | 8 | SELPLG | -0.62 | 0.06 | 0.10 | 0.14 |
| 8 | 1 | 8 | SH3GL2 | -0.80 | 0.06 | -0.13 | 0.33 |
| 8 | 1 | 8 | SH3RF2 | -1.34 | -0.19 | 0.36 | -0.08 |
| 8 | 1 | 8 | SHC4 | -0.61 | 0.33 | 0.28 | 0.27 |
| 8 | 1 | 8 | SHISA9 | -0.87 | 0.08 | -0.06 | -0.50 |
| 8 | 1 | 8 | SIX4 | -0.62 | 0.10 | -0.10 | -0.25 |
| 8 | 1 | 8 | SKA1 | -0.75 | -0.24 | -0.20 | -0.06 |
| 8 | 1 | 8 | SLC35F3 | -1.49 | -0.16 | -0.24 | 0.01 |
| 8 | 1 | 8 | SLC6A6 | -1.03 | -0.23 | 0.21 | 0.11 |
| 8 | 1 | 8 | SLFN12 | -0.54 | 0.18 | 0.10 | 0.09 |
| 8 | 1 | 8 | SLIT3 | -1.01 | 0.16 | -0.02 | -0.07 |
| 8 | 1 | 8 | SMURF2 | -0.58 | -0.05 | 0.06 | 0.08 |
| 8 | 1 | 8 | SNX5 | -0.69 | -0.22 | -0.16 | 0.21 |
| 8 | 1 | 8 | SOGA2 | -0.61 | 0.03 | 0.02 | -0.08 |
| 8 | 1 | 8 | SOX3 | -0.90 | 0.08 | -0.36 | -0.43 |
| 8 | 1 | 8 | SPAG5 | -0.84 | -0.21 | -0.27 | -0.21 |
| 8 | 1 | 8 | SPC25 | -0.83 | -0.20 | -0.28 | 0.09 |
| 8 | 1 | 8 | SPCS3 | -0.96 | -0.16 | 0.22 | 0.10 |
| 8 | 1 | 8 | SPDL1 | -0.89 | -0.31 | -0.33 | -0.02 |
| 8 | 1 | 8 | SPEG | -0.93 | 0.23 | 0.44 | -0.14 |
| 8 | 1 | 8 | SPP1 | -1.60 | 0.75 | 0.30 | 0.46 |
| 8 | 1 | 8 | SRPX | -0.59 | 0.27 | 0.13 | 0.53 |
| 8 | 1 | 8 | SRPX2 | -0.73 | -0.05 | 0.01 | 0.12 |
| 8 | 1 | 8 | SSH1 | -0.95 | -0.30 | 0.13 | -0.22 |
| 8 | 1 | 8 | STARD4 | -0.76 | -0.19 | -0.22 | 0.29 |
| 8 | 1 | 8 | STK17A | -0.68 | 0.35 | 0.11 | -0.16 |
| 8 | 1 | 8 | SYNJ2 | -1.15 | 0.06 | 0.13 | 0.62 |
| 8 | 1 | 8 | SYNPO2L | -1.40 | 0.10 | 0.29 | 0.20 |
| 8 | 1 | 8 | SYTL5 | -2.08 | -0.06 | 0.10 | 0.71 |
| 8 | 1 | 8 | TACR1 | -0.61 | 0.17 | 0.04 | 0.08 |
| 8 | 1 | 8 | TBC1D9 | -0.50 | 0.10 | -0.26 | 0.13 |
| 8 | 1 | 8 | TDP1 | -0.65 | 0.13 | 0.05 | 0.06 |
| 8 | 1 | 8 | TGFBR1 | -0.57 | 0.15 | -0.09 | -0.23 |
| 8 | 1 | 8 | TGM2 | -0.88 | 0.04 | -0.19 | 0.04 |
| 8 | 1 | 8 | THBS1 | -1.34 | -0.40 | 0.12 | -0.58 |
| 8 | 1 | 8 | TM4SF18 | -0.63 | 0.07 | -0.08 | -0.12 |
| 8 | 1 | 8 | TMEM132B | -1.45 | 0.11 | 0.05 | -0.14 |
| 8 | 1 | 8 | TMEM71 | -0.63 | 0.25 | 0.02 | 0.04 |
| 8 | 1 | 8 | TMX3 | -0.61 | 0.24 | -0.19 | 0.22 |
| 8 | 1 | 8 | TNNC1 | -0.75 | 0.17 | -0.10 | 0.00 |
| 8 | 1 | 8 | TOM1L2 | -0.64 | 0.10 | 0.18 | 0.06 |
| 8 | 1 | 8 | TOP2A | -0.65 | -0.10 | -0.26 | 0.10 |
| 8 | 1 | 8 | TPM1 | -0.84 | -0.37 | 0.10 | -0.25 |
| 8 | 1 | 8 | TSPAN18 | -0.71 | 0.10 | -0.16 | 0.12 |
| 8 | 1 | 8 | TTK | -0.72 | -0.04 | -0.10 | 0.13 |
| 8 | 1 | 8 | UACA | -0.78 | -0.36 | 0.14 | 0.18 |
| 8 | 1 | 8 | UBE2S | -0.54 | -0.02 | -0.25 | 0.00 |
| 8 | 1 | 8 | UBE2T | -0.64 | -0.09 | -0.20 | -0.01 |
| 8 | 1 | 8 | UCP2 | -0.97 | 0.14 | -0.35 | -0.08 |
| 8 | 1 | 8 | UGCG | -0.58 | 0.00 | -0.05 | 0.21 |
| 8 | 1 | 8 | UXS1 | -0.65 | 0.05 | -0.17 | 0.11 |
| 8 | 1 | 8 | WEE1 | -0.66 | -0.16 | -0.10 | 0.04 |
